# Mechanism of Ir(ppy)_3_ Guest Exciton Formation with the Exciplex-Forming TCTA:TPBI Cohost within a Phosphorescent Organic Light-Emitting Diode Environment

**DOI:** 10.3390/ijms23115940

**Published:** 2022-05-25

**Authors:** Jae Whee Park, Kwang Hyun Cho, Young Min Rhee

**Affiliations:** Department of Chemistry, Korea Advanced Institute of Science and Technology (KAIST), Daejeon 34141, Korea; pjwpeter@kaist.ac.kr (J.W.P.); jkhyun310@kaist.ac.kr (K.H.C.)

**Keywords:** phosphorescent OLEDs, exciplex-forming cohost, excitation energy transfer, charge transfer

## Abstract

Cohosts based on hole transporting and electron transporting materials often act as exciplexes in the form of intermolecular charge transfer complexes. Indeed, exciplex-forming cohosts have been widely developed as the host materials for efficient phosphorescent organic light-emitting diodes (OLEDs). In host–guest systems of OLEDs, the guest can be excited by two competing mechanisms, namely, excitation energy transfer (EET) and charge transfer (CT). Experimentally, it has been reported that the EET mechanism is dominant and the excitons are primarily formed in the host first and then transferred to the guest in phosphorescent OLEDs based on exciplex-forming cohosts. With this, exciplex-forming cohosts are widely employed for avoiding the formation of trapped charge carriers in the phosphorescent guest. However, theoretical studies are still lacking toward elucidating the relative importance between EET and CT processes in exciting the guest molecules in such systems. Here, we obtain the kinetics of guest excitation processes in a few trimer model systems consisting of an exciplex-forming cohost pair and a phosphorescent guest. We adopt the Förster resonance energy transfer (FRET) rate constants for the electronic transitions between excited states toward solving kinetic master equations. The input parameters for calculating the FRET rate constants are obtained from density functional theory (DFT) and time-dependent DFT. The results show that while the EET mechanism is important, the CT mechanism may still play a significant role in guest excitations. In fact, the relative importance of CT over EET depends strongly on the location of the guest molecule relative to the cohost pair. This is understandable as both the coupling for EET and the interaction energy for CT are strongly influenced by the geometric constraints. Understanding the energy transfer pathways from the exciplex state of cohost to the emissive state of guest may provide insights for improving exciplex-forming materials adopted in OLEDs.

## 1. Introduction

For constructing high-efficiency organic light-emitting diodes (OLEDs), exciplex materials that combine hole transporting materials (HTMs) and electron transporting materials (ETMs) to form intermolecular charge transfer (CT) complexes have been widely investigated [1,2,3,4,5,6,7,8,9,10,11,12,13,14,15,16]. Because exciplexes tend to display undesirable red-shifted and broadened emissions, they were sometimes considered to negatively affect OLED performance. However, one particular and apparent benefit of the broad spectrum was for the construction of white OLEDs (WOLEDs) with a high color rendering index. Therefore, exciplexes were found to be of utility as emitters in WOLEDs [12,13,14,15,16]. Besides being used as emitting materials, exciplexes can also be adopted as cohosts in the emissive layer (EML). The EMLs in OLEDs often consist of host and guest systems toward achieving the highest efficiency and device lifetime [17,18]. Because both charge carriers and excitation energies are transferred from the host to the guest to form ultimately emitting excitons in the guest, the role of host is actually very important for controlling the efficiency of OLEDs [19,20,21,22,23,24,25,26]. Various host materials have been studied for improving the performances and the lifetimes of phosphorescent OLEDs (PhOLEDs) [22,27]. Recently, exciplex-forming cohost materials have been widely developed for efficient PhOLEDs [1,2,3,4,5,6,7,8,9,10,11,28,29]. In more conventional PhOLEDs with a single host material, the host itself is responsible for both hole and electron transports in EML, and balancing the two mobilities is essentially nontrivial as tuning one property in the host inevitably touches the other. In contrast, with the exciplex-forming cohost, controlling the mobilities of holes and electrons separately becomes a relatively simple matter, and achieving efficient charge recombination in EML becomes more feasible [30,31]. For example, with better balanced mobilities with an exciplex-forming cohost, the recombination zone in EML can be spatially widened, leading to lower exciton density and to subsequent suppression of the triplet–triplet annihilation (TTA) process [7]. Indeed, light emission from a triplet exciton in PhOLEDs is a rather slow process, and suppressing the leaky TTA is important in maintaining the emission efficiency. Thus, using a cohost can become largely attractive with PhOLEDs. In addition, the triplet–polaron annihilation (TPA) process caused by the collision between a triplet exciton and a charged polaron can also be inhibited by suppressing the CT mechanism [7].

In the host–guest system of OLEDs, the guest is excited by two important competing mechanisms: excitation energy transfer (EET) and charge transfer (CT) [32,33,34,35]. While the excitons are first formed in the host and then directly transferred to the guest with the EET mechanism, a hole or an electron may be first trapped in the guest and then subsequently quenched by another electron or hole from the host with the CT mechanism. In general, the CT mechanism is dominant because phosphorescent guests act as hole or electron traps in conventional PhOLEDs. Therefore, the efficiency of conventional PhOLEDs decreases due to quenching processes such as TPA and TTA by trapped charge carriers and long-lived triplet excitons formed in the phosphorescent guest. In contrast, it has been experimentally reported that the EET mechanism is dominant in PhOLEDs with exciplex-forming cohosts [35,36,37,38,39]. The dominance has been ascribed to the good charge balance associated with the balanced mobilities and the formation of stable CT excitons in cohost pairs. Namely, the guest ionization is avoided through the direct EET from cohost to guest. However, this does not mean that the CT mechanism has a completely negligible contribution. In fact, investigating the details of the relative importance between EET and CT will benefit theoretical consideration, but such research on the mechanism of guest exciton formation in PhOLEDs with exciplex-forming cohosts has been limited. In this work, therefore, we will attempt such an examination. Thoroughly understanding the guest exciton formation mechanism will be crucial for improving the efficiency of PhOLEDs, as it will guide to designing more suitable host–guest systems.

**Figure 1:**
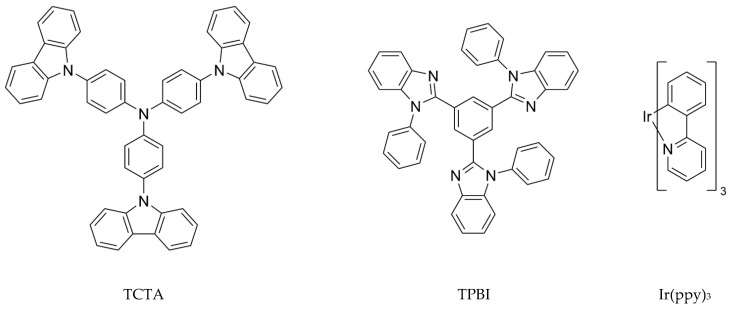
Chemical structures of cohost and guest molecules. TCTA and TPBI are, respectively, hole and electron transporting materials and constitute the exciplex-forming cohost. Ir(ppy)_3_ is a typical green phosphorescent guest.

The exemplary cohost and guest molecules used in this work are shown in Figure 1. For the exciplex-forming cohost, the pair of 4,4′,4″-tris(N-carbazolyl)triphenylamine (TCTA) and 1,3,5-tris(N-phenylbenzimidazole-2-yl)benzene (TPBI) was adopted [39]. For the phosphorescent guest, iridium (III) tris(2-phenylpyridine) (Ir(ppy)_3_) that serves as the green dopant was utilized [39]. TCTA and TPBI, respectively, act as HTM and ETM. We investigate the processes in which singlet and triplet CT excitons formed in the TCTA/TPBI pair are transferred to Ir(ppy)_3_. For this, we construct a few trimer model systems with TCTA, TPBI, and Ir(ppy)_3_ and calculate Förster resonance energy transfer (FRET) rate constants [40]. The input parameters for FRET rate constants are obtained from density functional theory (DFT) and time-dependent DFT (TDDFT) based on the Tamm–Dancoff approximation (TDA) [41]. With the calculated FRET rates, we investigate in detail how the guest is excited in each model system in singlet and triplet spin states.

## 2. Theory and Computational Details

### 2.1. Förster Theory

The kinetics of guest excitation processes were predicted by the Förster theory. The theory is based on the equilibrium Fermi’s golden rule approach with a second-order perturbation treatment of the electronic coupling between two involved electronic states [40,42,43,44]. Because the Förster theory is formulated for the weak coupling limit [45], it will work well for electronic transitions that occur in amorphous organic semiconductors such as OLEDs. In the Förster theory, the transfer rate from a state *i* to a state *j* is given by [46]

(1) $$k_{i\to j}=\frac{1}{N}\frac{2\pi }{\hbar }{\left|J_{ij}\right|}^{2}\int_{-\infty }^{\infty }f_{i}\left(\epsilon \right)a_{j}\left(\epsilon \right)d\epsilon$$

where $J_{ij}$ is the electronic coupling between the states *i* and *j*, with *N* denoting the area-normalization factor for the emission and the absorption spectra:

(2) $$N=\left[\int_{-\infty }^{\infty }f_{i}\left(\epsilon \right)d\epsilon \right]\times \left[\int_{-\infty }^{\infty }a_{j}\left(\epsilon \right)d\epsilon \right]$$

In this study, we assume Gaussian-shaped emission and absorption with the same site reorganization energies, which are homogeneously broadened:

(3) $$f_{i}\left(\epsilon \right)=\frac{1}{\sqrt{4\pi kT\lambda }}\exp\left[-\frac{{\left(\epsilon -{\overset{¯}{E}}_{i}+2\lambda \right)}^{2}}{4kT\lambda }\right]$$

(4) $$a_{j}\left(\epsilon \right)=\frac{1}{\sqrt{4\pi kT\lambda }}\exp\left[-\frac{{\left(\epsilon -{\overset{¯}{E}}_{j}\right)}^{2}}{4kT\lambda }\right]$$

where ${\overset{¯}{E}}_{i}$ and ${\overset{¯}{E}}_{j}$ are the vertical excitation energies of the two states, and *λ* is the reorganization energy. In Equation (3), 2*λ* in the numerator reflects the Stokes shift of the donor emission. With these, the FRET rate constant between the pair of states is calculated as

(5) $$k_{i\to j}=\frac{\sqrt{\pi }}{\hbar \sqrt{2kT\lambda }}{\left|J_{ij}\right|}^{2}\exp\left[-\frac{1}{2kT\lambda }{\left(\lambda -\frac{{\overset{¯}{E}}_{i}-{\overset{¯}{E}}_{j}}{2}\right)}^{2}\right]$$

The overall kinetics was described in terms of a master equation of the form

(6) $${\dot{P}}_{i}\left(t\right)=\underset{j\neq i}{\sum }\left[-k_{i\to j}P_{i}\left(t\right)+k_{j\to i}P_{j}\left(t\right)\right]$$

where *P_i_*(*t*) is the population of electronic state *i* at time *t*, such that $\sum_{i}P_{i}\left(t\right)=1$ is satisfied, and $k_{i\to j}$ is the FRET rate constant for the electronic transition from state *i* to state *j*.

### 2.2. Computational Details

The singlet ground state geometries of TCTA, TPBI, and Ir(ppy)_3_ were optimized with the B3LYP functional [47,48] using the 6-31G* basis set. For the Ir atom in Ir(ppy)_3_, the effective core potential in conjunction with the LACVP* basis set [49] was adopted. The trimer model systems were constructed by combining the optimized B3LYP geometries of the three molecules. Toward describing the complex, the *ω*B97X-D3 dispersion-corrected functional was employed instead for better describing noncovalent interactions. Toward describing the intermolecular CT state energies of the TCTA/TPBI complex, the optimally tuned (OT) range-separated hybrid functional OT-*ω*B97X-D3 was used. The range-separation parameter *ω* was tuned for this complex by minimizing the following expression [50,51,52,53]:

(7) $$J\left(\omega \right)=\left|E_{\mathrm{HOMO}}\left(\omega \right)+\mathrm{IP}\left(\omega \right)\right|+\left|E_{\mathrm{LUMO}}\left(\omega \right)+\mathrm{EA}\left(\omega \right)\right|$$

where, of course, *E*_HOMO_ and *E*_LUMO_ denote the energies of the highest occupied and lowest unoccupied molecular orbitals, and IP and EA are, respectively, the vertical first ionization potential and the electron affinity of the TCTA/TPBI complex. During optimizations in *ω*, we considered steps of 0.001 bohr^−1^. Because the energies of CT states are sensitive to the solid environment in amorphous organic semiconductors, the TDA-TDDFT calculations [54] of singlet and triplet excited states were performed employing the OT-*ω*B97X-D3 functional in the presence of a conductor-like polarizable continuum model (CPCM) with a dielectric constant of 3.0. For excited state calculations, the *ω* value was tuned in the presence of CPCM, which will be referred to as *ω*(CPCM). The electronic couplings between the excited states within the trimer model systems were obtained in the gas-phase via the BoysOV localization [55] starting from the TDA-TDDFT results with the OT-*ω*B97X-D3 functional. For the calculations of the electronic couplings, the *ω* value was re-tuned in the gas phase. We will refer to this *ω* value as *ω*(vac). All quantum chemical calculations were performed with Q-Chem 5.1 [56].

## 3. Results and Discussion

### 3.1. Trimer Model Systems

#### 3.1.1. Construction of TCTA/TPBI Complex

**Figure 2:**
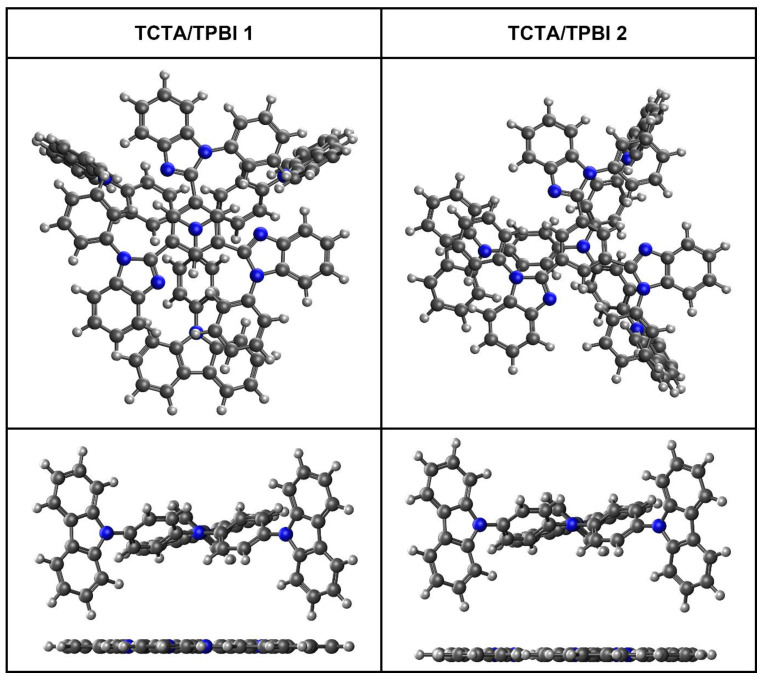
Illustrations of two different face-on TCTA/TPBI complexes.

In a solid film such as EML, molecules can randomly arrange themselves and there may exist various configurations with different distances and different relative orientations between molecules [57,58,59]. Molecules with disk-like shapes such as TCTA and TPBI will tend to form exciplexes of lower energy with larger intermolecular CT characters with face-on configurations [57]. The low-energy exciplex will enable efficient exciton confinement in the intermolecular CT state and limit energy leakage to a unimolecular decaying process within any constituent molecule, eventually allowing efficient EET to the guest [29]. For the face-on TCTA/TPBI complex, two configurations can be considered as illustrated in Figure 2.

**Figure 3:**
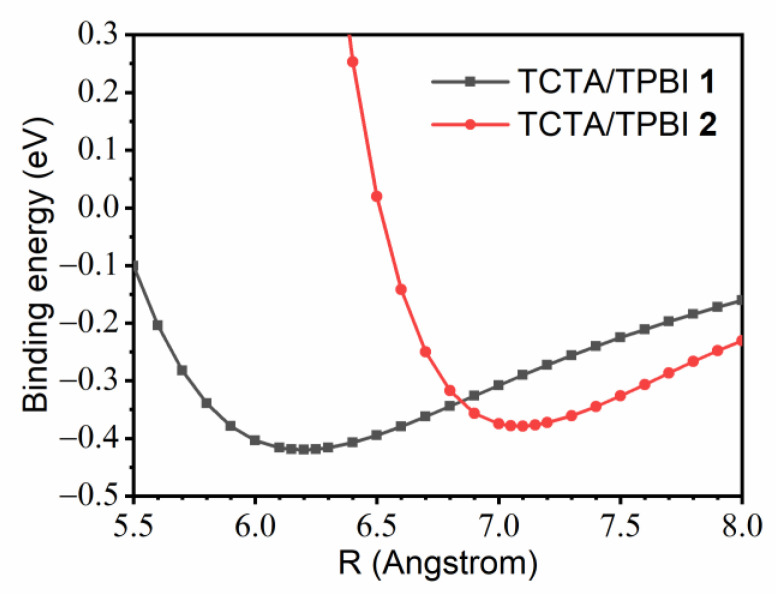
One-dimensional ground state potential energy surfaces of the TCTA/TPBI **1** and **2** as functions of the center-to-center distance between TCTA and TPBI.

To access the relative stabilities of the two potential configurations of the TCTA/TPBI complex, we obtained one-dimensional ground state potential energy surfaces (PESs) of the complex by scanning the center-to-center distance between the two moieties. We adopted the frozen-monomer approximation and did not allow relaxations of the monomers from their optimized B3LYP geometries [60]. The results are shown in Figure 3, and the binding energies of **1** and **2** obtained in this figure are 0.42 eV and 0.38 eV, respectively. While lifting the frozen-monomer approximation will surely further strengthen these binding energies, the relative difference between the two numbers will likely be similar. Because the difference (0.04 eV) is quite close to the thermal energy, both configurations and other similarly shaped complexes will form readily during the process of generating EML. The equilibrium distances between the two moieties are 6.20 Å for **1** and 7.10 Å for **2**. Thus, TCTA/TPBI **1** is more compact and has a slightly larger binding energy. For simplicity, therefore, when constructing the trimer model systems by further combining Ir(ppy)_3_, we employed **1** and fixed the TCTA-TPBI center-to-center distance at 6.20 Å.

#### 3.1.2. Construction of Trimer Model Systems

**Figure 4:**
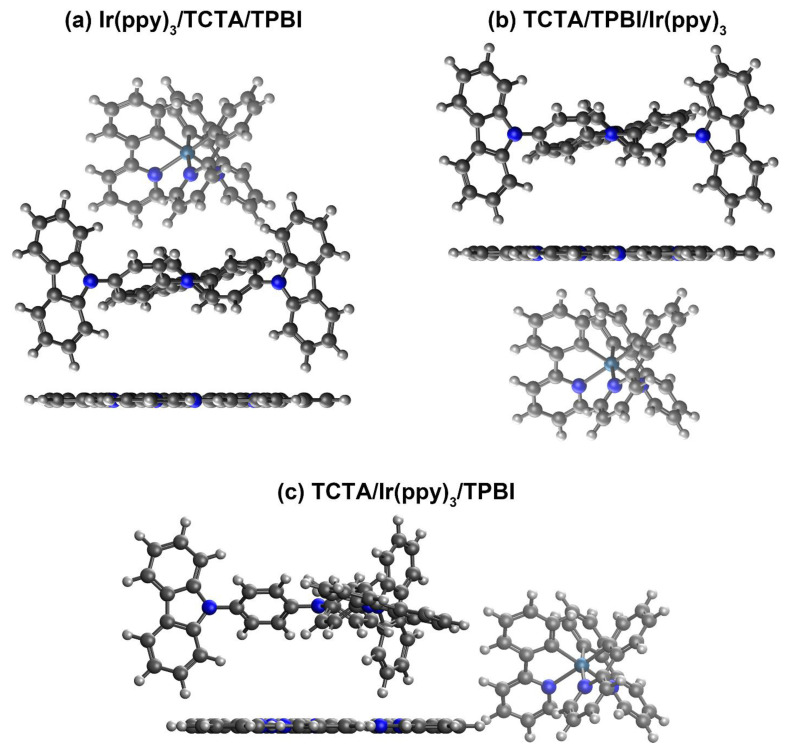
Illustrations of trimer model systems. For ease of distinction, Ir(ppy)_3_ is shown in lighter colors.

**Figure 5:**
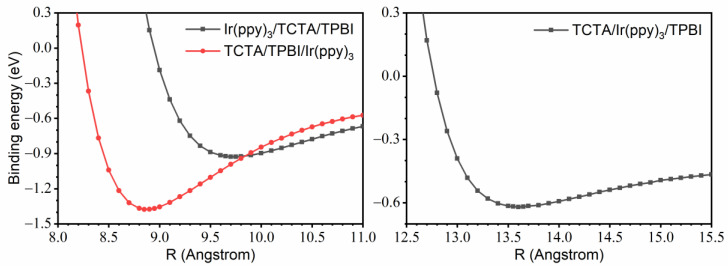
One-dimensional ground state potential energy surfaces of the trimer model systems as functions of the center-to-center distance between Ir(ppy)_3_ and the TCTA/TPBI complex.

As shown in Figure 4a,b, we considered two parallel configurations of trimer model systems by varying the locations of Ir(ppy)_3_ relative to the TCTA/TPBI complex, which will be respectively designated as Ir(ppy)_3_/TCTA/TPBI and TCTA/TPBI/Ir(ppy)_3_. In addition, another configuration with Ir(ppy)_3_ on the side of the TCTA/TPBI complex was also considered as depicted in Figure 4c. This third one will be designated as TCTA/Ir(ppy)_3_/TPBI. As in the case of the dimeric TCTA/TPBI complex, we first investigated the interaction patterns via one-dimensional ground state PESs of the trimer model systems. Again, the model systems were constructed using the separately optimized B3LYP ground state geometries of the Ir(ppy)_3_, TCTA, and TPBI and the PESs with the *ω*B97X-D3 functional were scanned with the frozen-monomer approach. As shown in Figure 5, the equilibrium center-to-center distances between the Ir(ppy)_3_ moiety and the TCTA/TPBI complex were found to be 9.75 Å for Ir(ppy)_3_/TCTA/TPBI, 8.85 Å for TCTA/TPBI/Ir(ppy)_3_, and 13.6 Å for TCTA/Ir(ppy)_3_/TPBI. In the following, we will describe the mechanisms of guest exciton formation in these three trimer configurations.

### 3.2. Excited States of Trimer Model Systems

Before charge recombination takes place in EML, electrons and holes are transported through TPBI and TCTA, respectively. In this work, we assumed that the initial state corresponded to a CT excited state where the positive and the negative charges are located in the TCTA and the TPBI moieties, respectively, and then investigated the potential pathways that may commence from this initial state. Of course, the considered final state was a local exciton state formed in the Ir(ppy)_3_ unit. Because singlet and triplet excitons are generated with a ratio of 1:3 under electrical excitation, both singlet and triplet states should be considered when studying the mechanism of guest exciton formation. The singlet states of the three trimer models will be denoted as ^1^(Ir(ppy)_3_/TCTA/TPBI), ^1^(TCTA/TPBI/Ir(ppy)_3_), and ^1^(TCTA/Ir(ppy)_3_/TPBI), and the triplet states will be marked similarly.

#### 3.2.1. Energies of Singlet and Triplet Excited States

**Figure 6:**
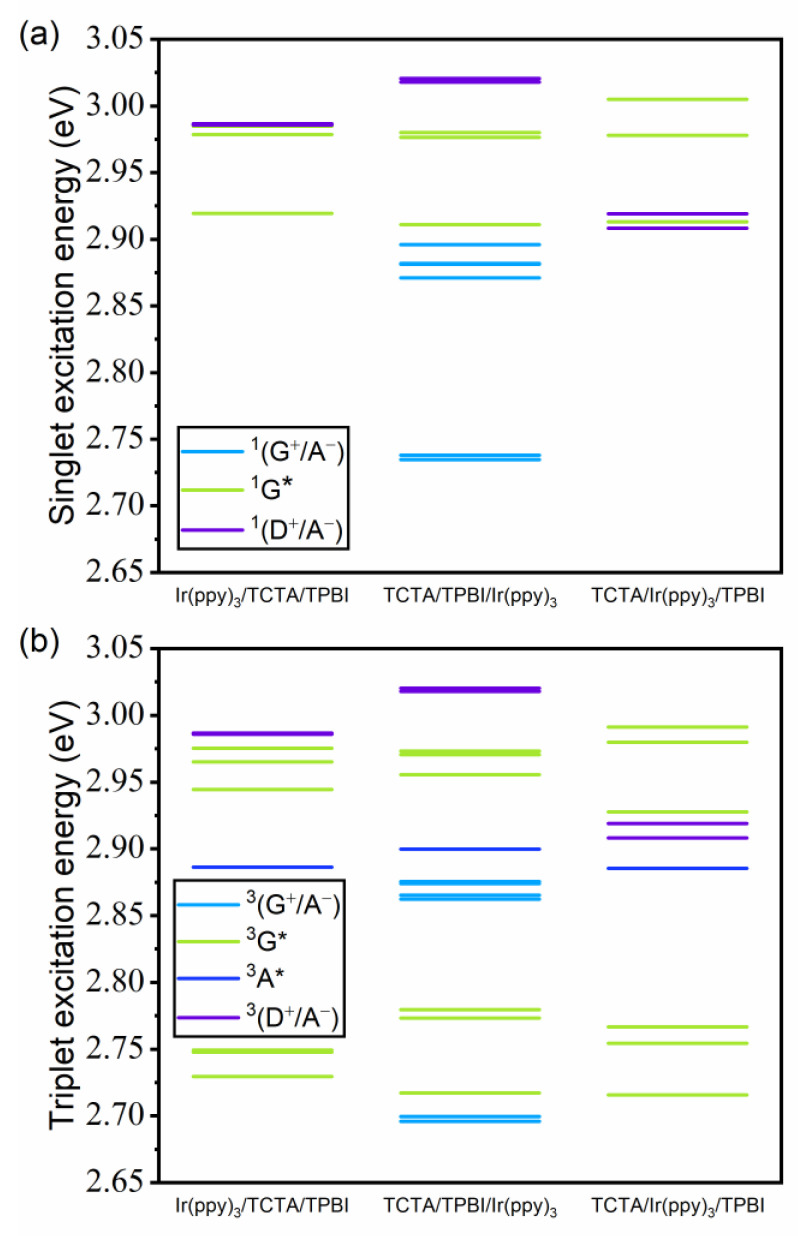
(**a**) Singlet and (**b**) triplet excited state energies of the three trimer model systems. In the legend, the three moieties are designated in a scheme of TCTA (electron donor) as D, TPBI (electron acceptor) as A, and Ir(ppy)_3_ (guest) as G. For example, ^3^(G^+^/A^−^) shown in light blue represents a triplet CT state with an electron migrated from Ir(ppy)_3_ to TPBI, and ^3^G* in green represents a triplet locally excited state of Ir(ppy)_3_.

The energies of singlet and triplet excited states of the three trimer model systems derived at the TDA-TDDFT/*ω*(CPCM)/CPCM level are shown in Figure 6, where we have color-coded the excitation characters together with simplified designations of the three constituting moieties. (See Tables S1 and S2 in the Supporting Information (SI) for the numerical values.) In the singlet manifold, the locally excited (LE) states of the donor and the acceptor (^1^D* and ^1^A*) are not considered because their energies (3.7 and 3.6 eV) are much higher compared to other low-lying states. As shown in Figure 6a, only the guest LE states (^1^G*) are below the lowest ^1^(D^+^/A^−^) in ^1^(Ir(ppy)_3_/TCTA/TPBI). In contrast, with ^1^(TCTA/TPBI/Ir(ppy)_3_), we can see that the guest-to-acceptor CT states, namely ^1^(G^+^/A^−^) states, lie below the lowest ^1^(D^+^/A^−^). Because Ir(ppy)_3_ and TPBI molecules are spatially quite close to each other in ^1^(TCTA/TPBI/Ir(ppy)_3_) and because the HOMO-LUMO gap involving the two molecules is rather small, as shown in Figure S1, the lowest ^1^(G^+^/A^−^) state can actually be lower in energy than the lowest ^1^(D^+^/A^−^) state. With ^1^(TCTA/Ir(ppy)_3_/TPBI), the energies of the ^1^(D^+^/A^−^) states are lower than those in the other two systems, as the ^1^(D^+^/A^−^) states are stabilized due to the increase in CT character (see Table S3). Moreover, there are no ^1^(G^+^/A^−^) states lying below the lowest ^1^(D^+^/A^−^) state due to the weak interaction between guest and acceptor. Within the triplet manifold shown in Figure 6b, the lowest ^3^(G^+^/A^−^) is still below the lowest ^3^(D^+^/A^−^) state in ^3^(TCTA/TPBI/Ir(ppy)_3_). One striking difference in comparison with the singlet manifold is the fact that an acceptor LE state (^3^A*) is quite low in energy and lies below the lowest ^3^(D^+^/A^−^) state, whereas donor LE states (^3^D*) are quite high in energy, with the lowest one at 3.2 eV.

In Figure 6, we note that no donor-to-guest CT states, namely no (D^+^/G^−^) states, show up. Interestingly, the (D^+^/G^−^) state was observed experimentally in the form of blue-shifted interface exciplex emission [61], and one may wonder why it is not found in our model complexes. As a matter of fact, the experimentally observed CT state was not formed in EML, but in an interface region between the exciton blocking layer (EBL) and EML. The CT state was formed between TCTA from EBL and Ir(ppy)_3_ from EML, and the blue-shifted interface emission was observed when the recombination zone was at the EBL/EML interface with the charge carriers accumulating at the interface. We actually found that the ^1^(D^+^/G^−^) state energy is 3.4 eV, which is lower than the ^1^D* state energy (3.7 eV). Therefore, if only TCTA and Ir(ppy)_3_ were considered, the ^1^(D^+^/G^−^) state would also get involved. However, in the EML containing TPBI (=A) as well as TCTA and Ir(ppy)_3_, the (D^+^/G^−^) state cannot participate importantly for emission as it will quickly decay to (D^+^/A^−^) at lower energy.

#### 3.2.2. Electronic Couplings between the Excited States

The findings in the above section suggest that there may be many additional states that can get involved toward EET between the cohost CT state (D^+^/A^−^) and guest LE state (G*). Even though we did not consider the triplet–singlet mixing effects [62,63] of Ir(ppy)_3_ in obtaining the energies of G* states, doing so will not likely change this aspect. In addition, the energetic structure strongly depends on how the complex is configured in space. Thus, it will be interesting to inspect the nature of the kinetic processes that will occur with the trimer models. Toward this end, obtaining the electronic coupling elements between the involved electronic states is crucial.

**Figure 7:**
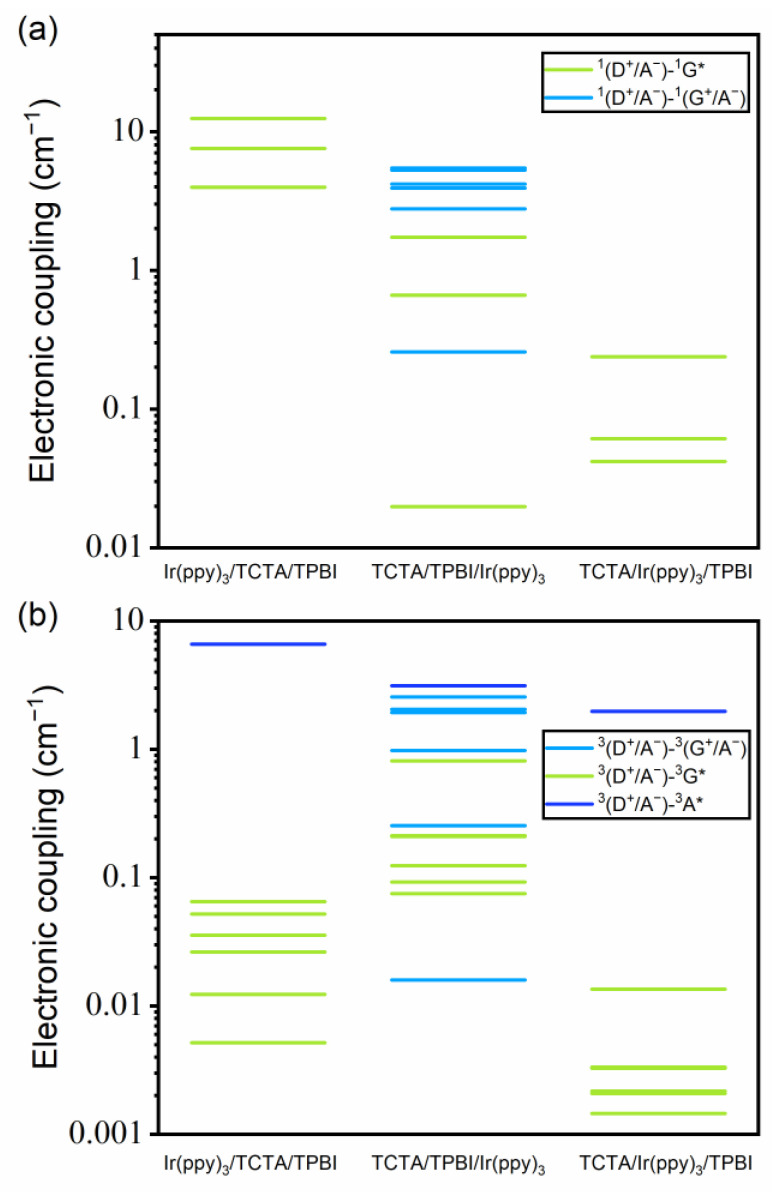
Pictorial representations of the electronic couplings (**a**) between the lowest ^1^(D^+^/A^−^) state and other singlet excited states, and (**b**) between the lowest ^3^(D^+^/A^−^) state and other triplet excited states.

Figure 7 displays the electronic couplings between the lowest cohost CT states and other excited states in the singlet and the triplet manifolds. The numerical values are listed in Tables S4–S9. Here, the (D^+^/A^−^)–G* electronic coupling is related to EET from the TCTA/TPBI complex to Ir(ppy)_3_. On the other hand, the (D^+^/A^−^)–(G^+^/A^−^) coupling drives the hole transfer (HT) from TCTA to Ir(ppy)_3_, while the (D^+^/A^−^)–A* coupling drives HT from TCTA to TPBI. In the singlet case (Figure 7a), while the largest electronic couplings are related to the ^1^(D^+^/A^−^)–^1^G* linked to the guest excitation via the EET mechanism with ^1^(Ir(ppy)_3_/TCTA/TPBI) and ^1^(TCTA/Ir(ppy)_3_/TPBI), the largest electronic coupling with ^1^(TCTA/TPBI/Ir(ppy)_3_) is with the ^1^(D^+^/A^−^)–^1^(G^+^/A^−^) coupling related to the CT mechanism. In addition, because the largest (D^+^/A^−^)–G* coupling with ^1^(TCTA/Ir(ppy)_3_/TPBI) is two orders of magnitude smaller than with ^1^(Ir(ppy)_3_/TCTA/TPBI), transferring singlet excitons directly from the TCTA/TPBI complex to Ir(ppy)_3_ will likely be extremely slow with ^1^(TCTA/Ir(ppy)_3_/TPBI). In any case, in the case of the singlet manifold, the dominant electronic coupling that determines the mechanism of guest exciton formation depends on the location of Ir(ppy)_3_ relative to the TCTA/TPBI complex.

Interestingly, the situation in the triplet manifold is quite different. As shown in Figure 7b, the ^3^(D^+^/A^−^)–^3^A* coupling is two orders of magnitude larger than the ^3^(D^+^/A^−^)–^3^G* electronic coupling with ^3^(Ir(ppy)_3_/TCTA/TPBI) and ^3^(TCTA/Ir(ppy)_3_/TPBI). Thus, we can imagine that transferring triplet excitons directly from the TCTA/TPBI complex to Ir(ppy)_3_ may be difficult with these trimer configurations. With ^3^(TCTA/TPBI/Ir(ppy)_3_), the largest ^3^(D^+^/A^−^)–^3^A* and ^3^(D^+^/A^−^)–^3^(G^+^/A^−^) couplings are less than one order magnitude larger than the largest ^3^(D^+^/A^−^)–^3^G* coupling. Thus, in the triplet state, it is conceivable that excited Ir(ppy)_3_ may readily form by direct cohost-to-guest EET, but additional pathways other than this one may still participate. Because both the state-to-state coupling and the associated energy difference affect the speed of interconversion between the two states, directly inspecting the kinetics will be a straightforward way of deciding the relative importance of diverse potential pathways.

### 3.3. Kinetics of Guest Excitation Processes

The kinetics of guest excitation processes in the trimer model systems were described in terms of the master equation (Equation (6)) using the FRET rate constants (Equation (5)) for the electronic transitions between the excited states. The calculated FRET rate constants are listed in Tables S10–S15. To describe the overall kinetics, we also included the intersystem crossing (ISC) rate (*k*_ISC_ = 5.7 × 10^12^ s^−1^) [64] and the radiative decay rate (*k*_r_ = 7.94 × 10^5^ s^−1^) [65] of Ir(ppy)_3_ in the master equation. In addition, spin conversion was not considered except for the ISC of Ir(ppy)_3_.

**Figure 8:**
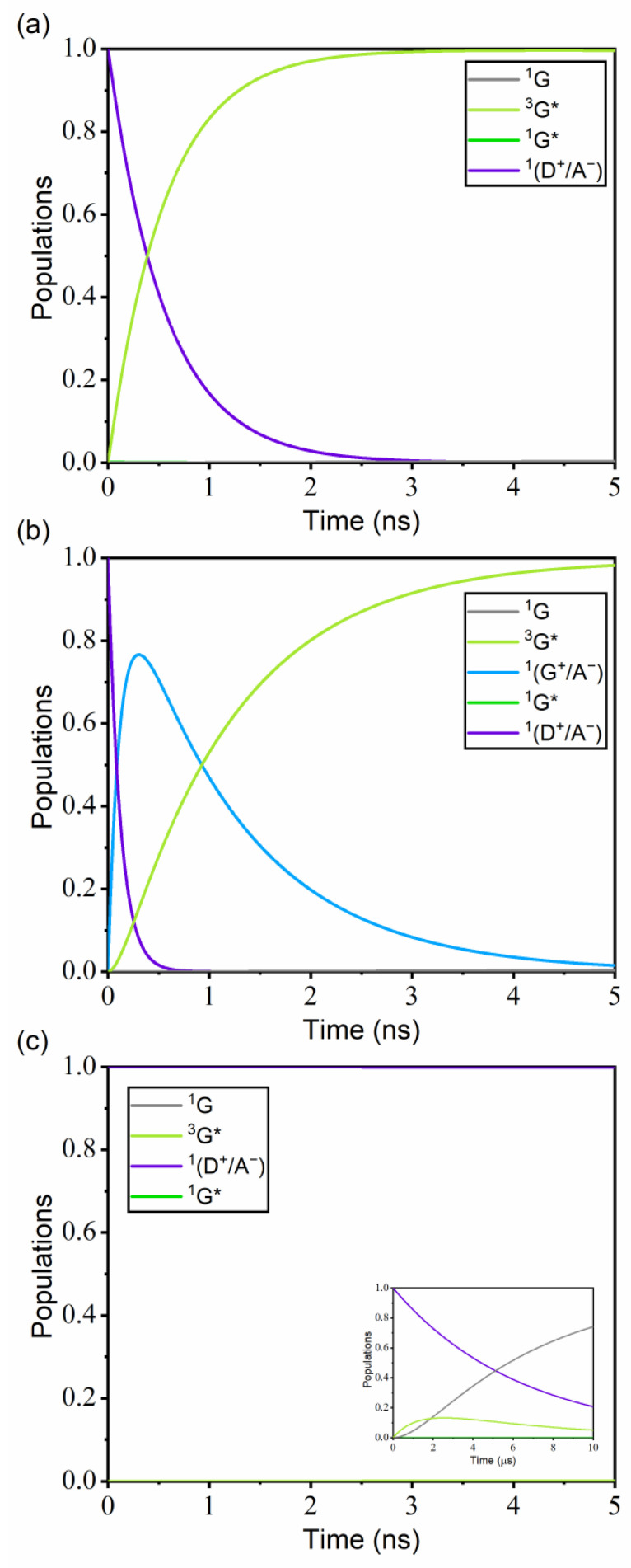
Population changes of the considered electronic states for (**a**) ^1^(Ir(ppy)_3_/TCTA/TPBI), (**b**) ^1^(TCTA/TPBI/Ir(ppy)_3_), and (**c**) ^1^(TCTA/Ir(ppy)_3_/TPBI) with the assumption that the initiating state is the lowest ^1^(D^+^/A^−^) state. Inset in (**c**) shows the data in the long-time limit. Except the ground state ^1^G, the population of each electronic state represents the sum of all populations of the states belonging to that type of electronic state. For example, the ^1^G* population includes all singlet excited populations of G. The considered electronic states are listed in Table S1. Rate constants were calculated with the Förster theory at 300 K, using the reorganization energy of 2000 cm^−1^.

Figure 8a shows the population changes in time for ^1^(Ir(ppy)_3_/TCTA/TPBI) when the initial excitation is in the cohost CT state, ^1^(D^+^/A^−^). The ^1^(D^+^/A^−^) population starts at unity and decays to zero by ~3 ns due to the fast EET from TCTA/TPBI to Ir(ppy)_3_. Because the *k*_ISC_ of Ir(ppy)_3_ is three orders of magnitude faster than the ^1^(D^+^/A^−^) → ^1^G* rate, the ISC process of the ^1^G* → ^3^G* transition takes place immediately after the ^1^G* formation. On the other hand, Figure 8b shows the population changes of ^1^(TCTA/TPBI/Ir(ppy)_3_) with the initial excitation in the same ^1^(D^+^/A^−^) state. The fact that the ^1^(G^+^/A^−^) population reaches a peak at ~0.3 ns implies that HT from TCTA to Ir(ppy)_3_ occurs quite rapidly. After HT, the remaining electron in TPBI transfers to Ir(ppy)_3_ to recombine with the hole in Ir(ppy)_3_. Therefore, in ^1^(TCTA/TPBI/Ir(ppy)_3_), the exciton in Ir(ppy)_3_ is formed sequentially by the CT mechanism with the hole transfer first and then the electron transfer next, whereas in ^1^(Ir(ppy)_3_/TCTA/TPBI), the exciton in Ir(ppy)_3_ is formed by the EET mechanism. For ^1^(TCTA/Ir(ppy)_3_/TPBI), the population changes with the initial excitation in the same ^1^(D^+^/A^−^) state indicate that the singlet CT exciton of the TCTA/TPBI complex transfers to Ir(ppy)_3_ by EET but very slowly due to the very weak ^1^(D^+^/A^−^)–^1^G* coupling (Figure 8c). Therefore, the contribution to emission by this non-parallel configuration will be negligible.

**Figure 9:**
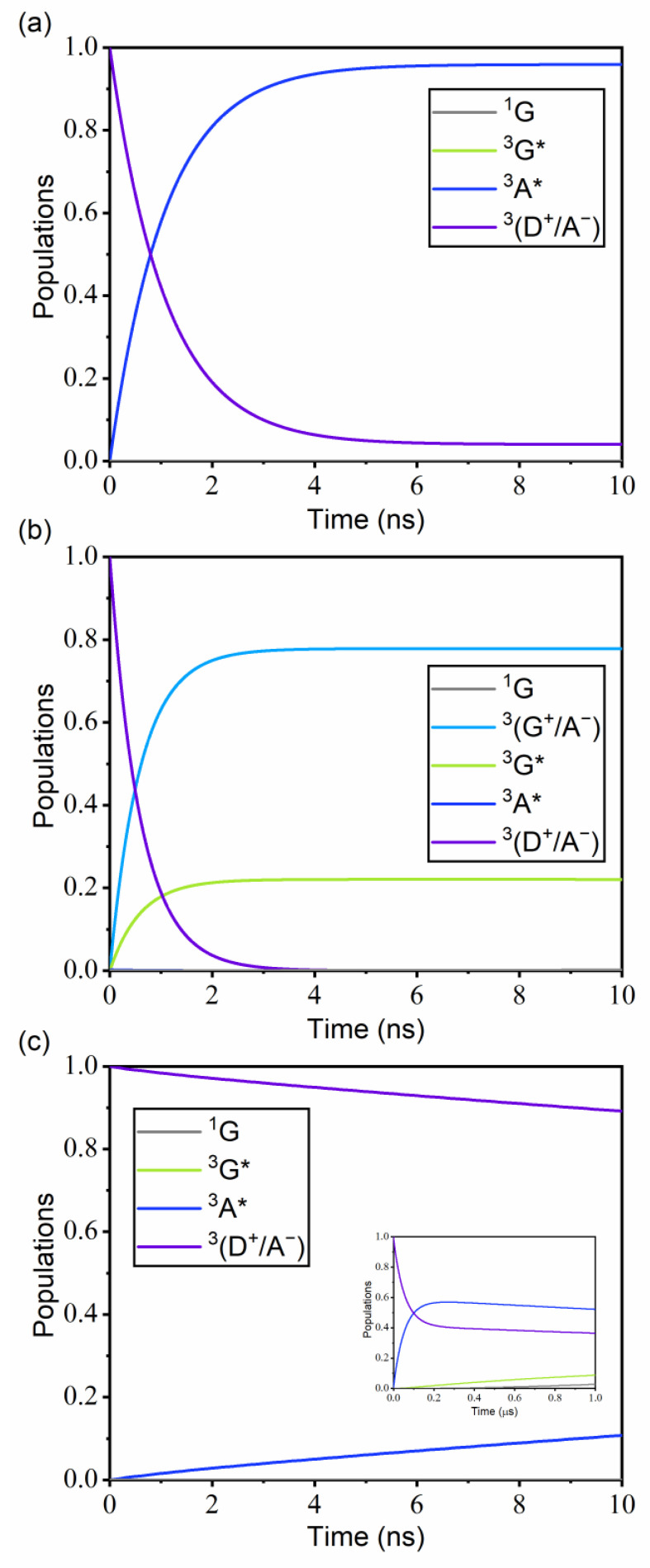
Population changes of the considered electronic states for (**a**) ^3^(Ir(ppy)_3_/TCTA/TPBI), (**b**) ^3^(TCTA/TPBI/Ir(ppy)_3_), and (**c**) ^3^(TCTA/Ir(ppy)_3_/TPBI) with the assumption that the initiating state is the lowest ^3^(D^+^/A^−^) state. Inset in (**c**) shows the data in the long-time limit. Each excited population is a sum of sub-populations as described in Figure 8. The considered electronic states are listed in Table S2. Rate constants were calculated with the Förster theory at 300 K, using the reorganization energy of 2000 cm^−1^.

Similarly, Figure 9a shows the population progressions in ^3^(Ir(ppy)_3_/TCTA/TPBI) when the initial excitation is with the triplet CT ^3^(D^+^/A^−^) state. The early decay of the ^3^(D^+^/A^−^) population is due to HT from the TCTA unit to TPBI. Because the ^3^(D^+^/A^−^) → ^3^A* rate is three orders of magnitude faster than the ^3^(D^+^/A^−^) → ^3^G* rate, the exciton is practically not formed in Ir(ppy)_3_ but in TPBI. In addition, because the ^3^A* → ^3^G* rate is also very slow at 2.44 × 10^2^ s^−1^ primarily due to the long distance between the TPBI and the Ir(ppy)_3_ moieties in ^3^(Ir(ppy)_3_/TCTA/TPBI), it takes a long time for the TPBI exciton to transfer to Ir(ppy)_3_ by EET. This is consistent with the experimental report that the energy can leak from the exciplex CT state to the triplet state of TPBI because the triplet state energy is lower than that of the exciplex [29]. In this energy leakage to TPBI, the magnitude of the ^3^(D^+^/A^−^)–^3^A* coupling, which is two orders larger than the ^3^(D^+^/A^−^)–^3^G* coupling, is actually the key. If the ^3^(D^+^/A^−^)–^3^G* coupling were much larger instead, ^3^G* would only slowly leak to ^3^A* and the emission from ^3^G* would not be lost. In any case, from the results, we can infer that the emission within ^3^(Ir(ppy)_3_/TCTA/TPBI) is relatively unimportant as the guest excitation pathways are kinetically blocked due to triplet energy leakage into TPBI.

Figure 9b displays the population changes with ^3^(TCTA/TPBI/Ir(ppy)_3_) with the same initial excitation ^3^(D^+^/A^−^). In this case, the ^3^(D^+^/A^−^) population decays essentially to zero by ~4 ns, and the ^3^(G^+^/A^−^) and ^3^G* populations rise almost simultaneously. In addition, the ^3^(G^+^/A^−^) and ^3^G* states rapidly equilibrate with each other. The energy difference between the two triplet states obtained by TDA-TDDFT is very small (0.02 eV), as already shown in Figure 6b. Because this small energy difference is within the TDA-TDDFT error range for triplet excitation energies [66], the correct state ordering between the two may be reversed. However, as long as the two states are close enough, the correct ordering is not actually important. This is because the two states are coupled quite strongly (−177 cm^−1^) and because the radiative decay of ^3^G* → ^1^G is relatively efficient. Namely, ^3^(G^+^/A^−^) will quickly equilibrate with ^3^G* and decay by the targeted emission (^3^G* → ^1^G). See Figure S2 for the long-time behavior.

Finally, the population changes with ^3^(TCTA/Ir(ppy)_3_/TPBI) shown in Figure 9c display similar trends with ^3^(Ir(ppy)_3_/TCTA/TPBI). Because the ^3^(D^+^/A^−^) → ^3^A* rate is three orders of magnitude faster than the ^3^(D^+^/A^−^) → ^3^G* rate, the exciton is not actually formed in Ir(ppy)_3_ but gets trapped in TPBI, just as in ^3^(Ir(ppy)_3_/TCTA/TPBI). Therefore, this configuration is again relatively unimportant toward investigating the emission pathways.

**Figure 10:**
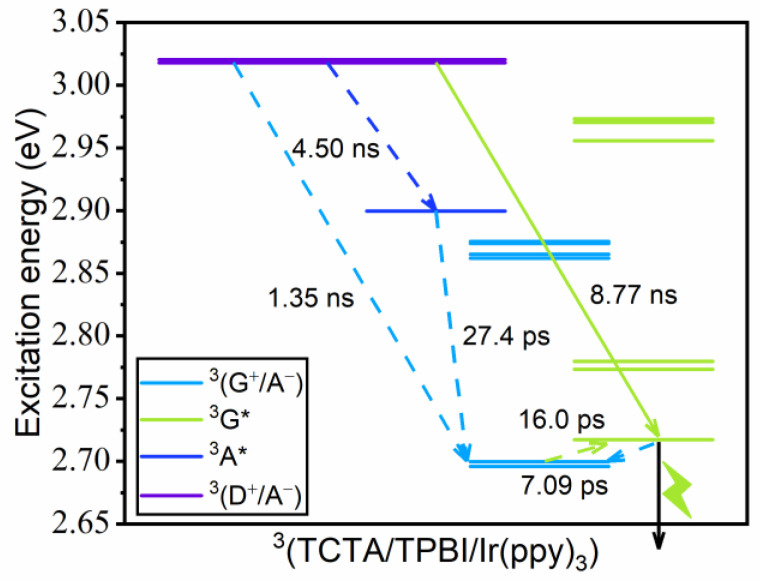
The time scales of important transitions in ^3^(TCTA/TPBI/Ir(ppy)_3_). Solid and dashed lines indicate EET and CT processes, respectively.

In Figure 10, we compare the time scales associated with some important transitions toward examining the details of the guest exciton formation mechanism in ^3^(TCTA/TPBI/Ir(ppy)_3_). The decay of the initial ^3^(D^+^/A^−^) population can be decomposed into three contributions, namely relaxations into ^3^(G^+^/A^−^), ^3^A*, and ^3^G* states with the time constants of 1.35 ns, 4.50 ns, and 8.77 ns. Thus, ^3^(D^+^/A^−^) relaxes predominantly into ^3^(G^+^/A^−^), which is associated with HT from TCTA to Ir(ppy)_3_. The ^3^A* population generated by ^3^(D^+^/A^−^) → ^3^A* mainly decays to ^3^(G^+^/A^−^) through a subsequent electron–hole separation. Radiative decay takes place from the emissive ^3^G* state, and as a result of the transition from ^3^(G^+^/A^−^) to ^3^G* driven by the large ^3^(G^+^/A^−^)–^3^G* coupling, all excitons are funneled to the emissive state. Of course, there will be additional loss mechanisms such as non-radiative decays to the ground state from all excited states that we did not consider in our kinetics, but their contributions will be minor. In the end, the triplet exciton of Ir(ppy)_3_ is formed by both the CT and the EET mechanisms. However, if we consider each transition separately, we can see that the triplet exciton of Ir(ppy)_3_ is predominantly generated by the CT mechanism. To further confirm this aspect observed with the Förster theory calculations, we have additionally performed mixed quantum-classical (MQC) simulations by adopting a simplified four-state system extracted from ^3^(TCTA/TPBI/Ir(ppy)_3_). Indeed, good correspondences were observed in comparison with Figure 9b. See Figure S3 and Table S16 for the details about these MQC simulations.

In single-host systems, because hole and electron mobilities are different in EML, holes or electrons are first trapped in guest molecules, and then these immobile charge carriers recombine with freely moving and oppositely charged carriers [35]. Therefore, OLEDs with a single host material may suffer from problems such as dissociation of guest molecules [67] or TPA initiated by the trapped charge carriers. In addition, because the recombination by carrier diffusion takes some time, the concentrations of trap sites may be rather high especially with high driving currents. In contrast, in a condition with cohosts, because G^+^ forms in a pairing manner with the neighboring negatively charged counterpart (A^−^) and the recombination is majorly driven by a practically geminate pair ((G^+^/A^−^) → G*), the concentration of the charged species will likely be low. Thus, the stability and/or the quenching issues will be less severe. In short, the enhanced performance with the exciplex-forming cohost will be majorly from the formation of charge pairs (G^+^/A^−^) instead of the unstable G^+^ alone, but not from complete avoidance of G^+^ via direct EET ((D^+^/A^−^) → G*). This also suggests that efforts to minimize the side pathways of single-host systems such as unwanted chemical reactions of ionic species and polaron-involving quenching should still be exercised even with exciplex-forming cohost systems.

By examining the kinetics of interconversions between diverse spin-electronic states, we can indeed provide insights toward designing high-efficiency exciplex-forming cohost PhOLEDs. For example, for attaining high efficiency, we need to increase the ratio of the EET mechanism to suppress triplet quenching processes such as TTA and TPA. Our results show that in the (TCTA/TPBI/Ir(ppy)_3_) case, the CT mechanism plays a more important role than the EET mechanism in transferring both singlet and triplet excitons of TCTA/TPBI to Ir(ppy)_3_ and (G^+^/A^−^) states are very importantly involved. Therefore, to hinder the formation of (G^+^/A^−^) states, reducing the interaction between Ir(ppy)_3_ and TPBI, namely G and A, may be attempted potentially by attaching bulky groups to TPBI toward increasing the contacting distance between them. In addition, because the guest excitation pathways are kinetically blocked due to triplet energy leakage to TPBI in ^3^(Ir(ppy)_3_/TCTA/TPBI), adopting a different electron transferring material with a higher triplet energy than TPBI may be beneficial. Finally, because the EET mechanism has higher significance in the singlet manifold, somehow encouraging triplet to singlet conversion within the host pair itself may deserve some consideration. In any case, by thoroughly understanding the guest excitation mechanism, it will be possible to design better EML materials for improving the efficiency of PhOLEDs with the exciplex-forming cohost.

Indeed, the exciplex system with the TCTA/TPBI complex was already adopted as a blue emitter for constructing WOLEDs [15]. In addition, a tactic of adopting multiple Ir-complexes in multiple emission layers was also reported for forming WOLEDs [68]. Thus, one can imagine diverse tactics of formulating WOLEDs with multiple EMLs, potentially with differing exciplexes and Ir-complexes in each layer. With good charge balances in each layer, charge trapping in any layer will be minimized.

## 4. Conclusions

In this work, we have investigated guest excitation pathways in a PhOLED system with Ir(ppy)_3_ as the guest and TCTA/TPBI pair as the exciplex-forming cohost. We have considered the kinetics of possible guest excitation processes after constructing three trimer model systems by varying the location of the Ir(ppy)_3_ moiety relative to the TCTA/TPBI complex. We found that the CT mechanism that generates an ionized form of the guest molecule still plays a significant role toward the final guest excitation, which is in stark contrast to the common belief that the cohost-to-guest direct EET that circumvents such ionization is the major path. In addition, we also showed that this relative importance of the CT mechanism over the EET mechanism depends strongly on the location of the guest molecule relative to the cohost pair. In the singlet case, the Ir(ppy)_3_ exciton is formed by the EET mechanism with the ^1^(Ir(ppy)_3_/TCTA/TPBI) configuration, whereas it is formed by the CT mechanism with ^1^(TCTA/TPBI/Ir(ppy)_3_). In the latter configuration, the ^1^(G^+^/A^−^) state, namely the CT state where the Ir(ppy)_3_/TPBI pair involves the charge migration, plays an important role as an intermediate state. In the triplet case, the location of Ir(ppy)_3_ still strongly affects the mechanism, but how it influences the guest excitation pathways is quite different from the singlet case. With the ^3^(Ir(ppy)_3_/TCTA/TPBI) configuration, the triplet exciton gets trapped in TPBI due to the strong coupling between the triplet CT state of TCTA/TPBI complex and the triplet state of TPBI. Although the ^3^G*, namely the emissive state of Ir(ppy)_3_, is lower in energy than the triplet state of TPBI, the trapped exciton in TPBI cannot migrate to Ir(ppy)_3_ because the coupling between the two states is very small, and thus the guest exciton formation is kinetically blocked in this configuration. On the other hand, with ^3^(TCTA/TPBI/Ir(ppy)_3_), the ^3^(G^+^/A^−^) CT state acts as an intermediate state toward forming the emissive state of Ir(ppy)_3_. Thus, the final guest excitation is funneled by transiently forming the hole-trapped Ir(ppy)_3_ and the dominant pathway can be classified as the CT mechanism. Unlike the conventional single host condition, the charge on the guest can be readily quenched by the adjacent counter ion; here, the negatively charged TPBI and ^3^(G^+^/A^−^) and ^3^G* exist in equilibrium throughout the emission. Additionally, regardless of spin states, the configuration with Ir(ppy)_3_ approaching the TCTA/TPBI stack from its side is less important with rather weak couplings for state switching in both singlet and triplet manifolds.

Consequently, tactics of avoiding related decay paths should still be exercised even with exciplex-forming cohosts, because the (G^+^/A^−^) CT state, in which a hole is formed in Ir(ppy)_3_, is still important during the guest excitation process. In addition, the relative position of Ir(ppy)_3_ to the TCTA/TPBI complex significantly affects the mechanism of guest excitation. Understanding the energy transfer pathways from the exciplex state of the cohost to the emissive state of the guest may provide insights for improving exciplex-forming materials adopted in OLEDs.

## Data Availability

Not applicable.

## Supplementary Materials

The following supporting information can be downloaded at: https://www.mdpi.com/article/10.3390/ijms23115940/s1.

## References

[1] Luo D., Liao C.-W., Chang C.-H., Tsai C.-C., Lu C.-W., Chuang T.C., Chang H.-H. (2020). Approach to fast screen the formation of an exciplex. J. Phys. Chem. C.

[2] Kim S.-Y., Jeong W.-I., Mayr C., Park Y.-S., Kim K.-H., Lee J.-H., Moon C.-K., Brütting W., Kim J.-J. (2013). Organic Light-Emitting diodes with 30% external quantum efficiency based on a horizontally oriented emitter. Adv. Funct. Mater..

[3] Park Y.-S., Lee S., Kim K.-H., Kim S.-Y., Lee J.-H., Kim J.-J. (2013). Exciplex-forming co-host for organic light-emitting diodes with ultimate efficiency. Adv. Funct. Mater..

[4] Lee J.Y. (2014). Mixed-host-emitting layer for high-efficiency organic light-emitting diodes. J. Inf. Disp..

[5] Lee S., Kim K.-H., Limbach D., Park Y.-S., Kim J.-J. (2013). Low roll-off and high efficiency orange organic light emitting diodes with controlled co-doping of green and red phosphorescent dopants in an exciplex forming co-host. Adv. Funct. Mater..

[6] Song W., Lee J.Y. (2017). Design strategy of exciplex host for extended operational lifetime. Org. Electron..

[7] Jung M., Lee J.Y. (2020). Exciplex hosts for blue phosphorescent organic light-emitting diodes. J. Inf. Disp..

[8] Lim H., Shin H., Kim K.-H., Yoo S.-J., Huh J.-S., Kim J.-J. (2017). An exciplex host for deep-blue phosphorescent organic light-emitting diodes. ACS Appl. Mater. Interfaces.

[9] Lee J.-H., Shin H., Kim J.-M., Kim K.-H., Kim J.-J. (2017). Exciplex-forming co-host-based red phosphorescent organic light-emitting diodes with long operational stability and high efficiency. ACS Appl. Mater. Interfaces.

[10] Shin H., Lee S., Kim K.-H., Moon C.-K., Yoo S.-J., Lee J.-H., Kim J.-J. (2014). Blue phosphorescent organic light-emitting diodes using an exciplex forming co-host with the external quantum efficiency of theoretical limit. Adv. Mater..

[11] Liu X., Yao B., Zhang Z., Zhao X., Zhang B., Wong W.-Y., Cheng Y., Xie Z. (2016). Power-efficient solution-processed red organic light-emitting diodes based on an exciplex host and a novel phosphorescent iridium complex. J. Mater. Chem. C.

[12] Liu B., Hu S., Zhang L., Xiao P., Huang L., Liu C. (2021). Blue molecular emitter-free and doping-free white organic light-emitting diodes with high color rendering. IEEE Electron Device Lett..

[13] Luo D., Xiao Y., Hao M., Zhao Y., Yang Y., Gao Y., Liu B. (2017). Doping-free white organic light-emitting diodes without blue molecular emitter: An unexplored approach to achieve high performance via exciplex emission. Appl. Phys. Lett..

[14] Luo D., Li X.-L., Zhao Y., Gao Y., Liu B. (2017). High-performance blue molecular emitter-free and doping-free hybrid white organic light-emitting diodes: An alternative concept to manipulate charges and excitons based on exciplex and electroplex emission. ACS Photonics.

[15] Wang L., Kou Z., Wang B., Zhou J., Lu Z., Li L. (2021). Realizing high efficiency/CRI/color stability in the hybrid white organic light emitting diode by manipulating exciton energy transfer. Opt. Mater..

[16] Xiao P., Huang J., Yu Y., Yuan J., Luo D., Liu B., Liang D. (2018). Recent advances of exciplex-based white organic light-emitting diodes. Appl. Sci..

[17] Tao Y., Yang C., Qin J. (2011). Organic host materials for phosphorescent organic light-emitting diodes. Chem. Soc. Rev..

[18] Chaskar A., Chen H.-F., Wong K.-T. (2011). Bipolar host materials: A chemical approach for highly efficient electrophosphorescent devices. Adv. Mater..

[19] Yeh S.-J., Wu M.-F., Chen C.-T., Song Y.-H., Chi Y., Ho M.-H., Hsu S.-F., Chen C.H. (2005). New dopant and host materials for Blue-Light-Emitting phosphorescent organic electroluminescent devices. Adv. Mater..

[20] Tokito S., Iijima T., Suzuri Y., Kita H., Tsuzuki T., Sato F. (2003). Confinement of triplet energy on phosphorescent molecules for highly-efficient organic blue-light-emitting devices. Appl. Phys. Lett..

[21] Nakanotani H., Masui K., Nishide J., Shibata T., Adachi C. (2013). Promising operational stability of high-efficiency organic light-emitting diodes based on thermally activated delayed fluorescence. Sci. Rep..

[22] Baldo M.A., Lamansky S., Burrows P.E., Thompson M.E., Forrest S.R. (1999). Very high-efficiency green organic light-emitting devices based on electrophosphorescence. Appl. Phys. Lett..

[23] Zhou X., Qin D.S., Pfeiffer M., Blochwitz-Nimoth J., Werner A., Drechsel J., Maennig B., Leo K., Bold M., Erk P. (2002). High-efficiency electrophosphorescent organic light-emitting diodes with double light-emitting layers. Appl. Phys. Lett..

[24] Adachi C., Baldo M.A., Thompson M.E., Forrest S.R. (2001). Nearly 100% internal phosphorescence efficiency in an organic light-emitting device. J. Appl. Phys..

[25] Scott J.C., Karg S., Carter S.A. (1997). Bipolar charge and current distributions in organic light-emitting diodes. J. Appl. Phys..

[26] Baldo M.A., Thompson M.E., Forrest S.R. (1999). Phosphorescent materials for application to organic light emitting devices. Pure Appl. Chem..

[27] Adachi C., Baldo M.A., Forrest S.R., Thompson M.E. (2000). High-efficiency organic electrophosphorescent devices with tris (2-phenylpyridine) iridium doped into electron-transporting materials. Appl. Phys. Lett..

[28] Zhang D., Cai M., Zhang Y., Bin Z., Zhang D., Duan L. (2016). Simultaneous enhancement of efficiency and stability of phosphorescent OLEDs based on efficient Förster energy transfer from interface exciplex. ACS Appl. Mater. Interfaces.

[29] Ban X., Sun K., Sun Y., Huang B., Jiang W. (2016). Enhanced electron affinity and exciton confinement in exciplex-type host: Power efficient solution-processed blue phosphorescent OLEDs with low turn-on voltage. ACS Appl. Mater. Interfaces.

[30] Hsiao C.-H., Chen Y.-H., Lin T.-C., Hsiao C.-C., Lee J.-H. (2006). Recombination zone in mixed-host organic light-emitting devices. Appl. Phys. Lett..

[31] Lee J., Lee J.-I., Lee J.Y., Chu H.Y. (2009). Enhanced efficiency and reduced roll-off in blue and white phosphorescent organic light-emitting diodes with a mixed host structure. Appl. Phys. Lett..

[32] Lane P.A., Palilis L.C., O’Brien D.F., Giebeler C., Cadby A.J., Lidzey D.G., Campbell A.J., Blau W., Bradley D.D.C. (2001). Origin of electrophosphorescence from a doped polymer light emitting diode. Phys. Rev. B.

[33] Wetzelaer G.A.H., Kuik M., Nicolai H.T., Blom P.W.M. (2011). Trap-assisted and Langevin-type recombination in organic light-emitting diodes. Phys. Rev. B.

[34] Peng Q., Gao N., Li W., Chen P., Li F., Ma Y. (2013). Investigation of energy transfer and charge trapping in dye-doped organic light-emitting diodes by magneto-electroluminescence measurement. Appl. Phys. Lett..

[35] Lee J.-H., Lee S., Yoo S.-J., Kim K.-H., Kim J.-J. (2014). Langevin and trap-assisted recombination in phosphorescent organic light emitting diodes. Adv. Funct. Mater..

[36] Kim K.H., Lee J.Y., Park T.J., Jeon W.S., Kennedy G.P., Kwon J.H. (2010). Small molecule host system for solution-processed red phosphorescent OLEDs. Synth. Met..

[37] Park Y.-S., Jeong W.-I., Kim J.-J. (2011). Energy transfer from exciplexes to dopants and its effect on efficiency of organic light-emitting diodes. J. Appl. Phys..

[38] Seo S., Shitagaki S., Ohsawa N., Inoue H., Suzuki K., Nowatari H., Yamazaki S. (2014). Exciplex-triplet energy transfer: A new method to achieve extremely efficient organic light-emitting diode with external quantum efficiency over 30% and drive voltage below 3 V. Jpn. J. Appl. Phys..

[39] Song W., Lee J.Y. (2015). Light emission mechanism of mixed host organic light-emitting diodes. Appl. Phys. Lett..

[40] Förster T. (1948). Zwischenmolekulare energiewanderung und fluoreszenz. Ann. Phys..

[41] Hirata S., Head-Gordon M. (1999). Time-dependent density functional theory within the Tamm–Dancoff approximation. Chem. Phys. Lett..

[42] Scholes G.D. (2003). Long-range resonance energy transfer in molecular systems. Annu. Rev. Phys. Chem..

[43] Wilkins D.M., Dattani N.S. (2015). Why quantum coherence is not important in the Fenna–Matthews–Olsen complex. J. Chem. Theory Comput..

[44] Ishizaki A., Fleming G.R. (2009). On the adequacy of the Redfield equation and related approaches to the study of quantum dynamics in electronic energy transfer. J. Chem. Phys..

[45] Renger T. (2009). Theory of excitation energy transfer: From structure to function. Photosynth. Res..

[46] Yang M., Fleming G.R. (2002). Influence of phonons on exciton transfer dynamics: Comparison of the Redfield, Förster, and modified Redfield equations. Chem. Phys..

[47] Beck A.D. (1993). Density-functional thermochemistry. III. The role of exact exchange. J. Chem. Phys..

[48] Lee C., Yang W., Parr R.G. (1988). Development of the Colle-Salvetti correlation-energy formula into a functional of the electron density. Phys. Rev. B.

[49] Hay P.J., Wadt W.R. (1985). Ab initio effective core potentials for molecular calculations. Potentials for K to Au including the outermost core orbitals. J. Chem. Phys..

[50] Garza A.J., Osman O.I., Wazzan N.A., Khan S.B., Asiri A.M., Scuseria G.E. (2014). A computational study of the nonlinear optical properties of carbazole derivatives: Theory refines experiment. Theor. Chem. Acc..

[51] Pandey L., Doiron C., Sears J.S., Brédas J.-L. (2012). Lowest excited states and optical absorption spectra of donor–acceptor copolymers for organic photovoltaics: A new picture emerging from tuned long-range corrected density functionals. Phys. Chem. Chem. Phys..

[52] Karolewski A., Stein T., Baer R., Kümmel S. (2011). Communication: Tailoring the optical gap in light-harvesting molecules. J. Chem. Phys..

[53] Stein T., Kronik L., Baer R. (2009). Reliable prediction of charge transfer excitations in molecular complexes using time-dependent density functional theory. J. Am. Chem. Soc..

[54] Runge E., Gross E.K.U. (1984). Density-functional theory for time-dependent systems. Phys. Rev. Lett..

[55] Subotnik J.E., Vura-Weis J., Sodt A.J., Ratner M.A. (2010). Predicting accurate electronic excitation transfer rates via Marcus theory with Boys or Edmiston− Ruedenberg localized diabatization. J. Phys. Chem. A.

[56] Shao Y., Gan Z., Epifanovsky E., Gilbert A.T.B., Wormit M., Kussmann J., Lange A.W., Behn A., Deng J., Feng X. (2015). Advances in molecular quantum chemistry contained in the Q-Chem 4 program package. Mol. Phys..

[57] Moon C.-K., Huh J.-S., Kim J.-M., Kim J.-J. (2018). Electronic structure and emission process of excited charge transfer states in solids. Chem. Mater..

[58] Hung W.-Y., Chiang P.-Y., Lin S.-W., Tang W.-C., Chen Y.-T., Liu S.-H., Chou P.-T., Hung Y.-T., Wong K.-T. (2016). Balance the carrier mobility to achieve high performance exciplex OLED using a triazine-based acceptor. ACS Appl. Mater. Interfaces.

[59] Angioni E., Chapran M., Ivaniuk K., Kostiv N., Cherpak V., Stakhira P., Lazauskas A., Tamulevičius S., Volyniuk D., Findlay N.J. (2016). A single emitting layer white OLED based on exciplex interface emission. J. Mater. Chem. C.

[60] Zhang C.-R., Sears J.S., Yang B., Aziz S.G., Coropceanu V., Brédas J.-L. (2014). Theoretical study of the local and charge-transfer excitations in model complexes of pentacene-C_60_ using tuned range-separated hybrid functionals. J. Chem. Theory Comput..

[61] Lee W.H., Kim D.H., Jesuraj P.J., Hafeez H., Lee J.C., Choi D.K., Bae T.-S., Yu S.M., Song M., Kim C.S. (2018). Improvement of charge balance, recombination zone confinement, and low efficiency roll-off in green phosphorescent OLEDs by altering electron transport layer thickness. Mater. Res. Express.

[62] Hedley G.J., Ruseckas A., Samuel I.D.W. (2008). Ultrafast luminescence in Ir(ppy)_3_. Chem. Phys. Lett..

[63] Gertsen A.S., Koerstz M., Mikkelsen K.V. (2018). Benchmarking triplet–triplet annihilation photon upconversion schemes. Phys. Chem. Chem. Phys..

[64] Kleinschmidt M., Wüllen C.V., Marian C.M. (2015). Intersystem-crossing and phosphorescence rates in fac-Ir*^III^*(ppy)_3_: A theoretical study involving multi-reference configuration interaction wavefunctions. J. Chem. Phys..

[65] de Vries X., Friederich P., Wenzel W., Coehoorn R., Bobbert P.A. (2019). Triplet exciton diffusion in metalorganic phosphorescent host-guest systems from first principles. Phys. Rev. B.

[66] Peach M.J.G., Williamson M.J., Tozer D.J. (2011). Influence of triplet instabilities in TDDFT. J. Chem. Theory Comput..

[67] de Moraes I.R., Scholz S., Lüssem B., Leo K. (2011). Role of oxygen-bonds in the degradation process of phosphorescent organic light emitting diodes. Appl. Phys. Lett..

[68] Liu B., Wang L., Tao H., Xu M., Zou J., Ning H., Peng J., Cao Y. (2017). Doping-free tandem white organic light-emitting diodes. Sci. Bull..

