# Supplementary material for: Mechanism of Ir(ppy)3 Guest Exciton Formation with the Exciplex-Forming TCTA:TPBI Cohost within a Phosphorescent Organic Light-Emitting Diode Environment
Source: Int J Mol Sci. 2022 May 25;23(11):5940. doi: 10.3390/ijms23115940 (PMC9180450; doi:10.3390/ijms23115940)
Supplement: Supplementary file 1 [file ijms-23-05940-s001.zip › ijms-1714824-supplementary.pdf]

Supporting Information for

Mechanism of $Ir(ppy)_3$ Guest Exciton Formation with the Exciplex-Forming TCTA:TPBI Cohost within a Phosphorescent Organic Light Emitting Diode Environment

Jae Whee Park, Kwang Hyun Cho, and Young Min Rhee*

Department of Chemistry, Korea Advanced Institute of Science and Technology (KAIST), Daejeon 34141, Korea

* E-mail: ymrhee@kaist.ac.kr

Table S1. Singlet excited state energies obtained by TDA-TDDFT/CPCM and the ωB97X-D3 functional with the optimized ω(CPCM) value.[a]

		E (eV)
^{1}(Ir(ppy)$_3$/TCTA/TPBI)	$^1G^*_1$	2.9193
	$^1G^*_2$	2.9785
	$^1G^*_3$	2.9849
	$^1(D^+/A^-)_1$	2.9858
	$^1(D^+/A^-)_2$	2.9867
^{1}(TCTA/TPBI/Ir(ppy)$_3$)	$^1(G^+/A^-)_1$	2.7348
	$^1(G^+/A^-)_2$	2.7380
	$^1(G^+/A^-)_3$	2.8710
	$^1(G^+/A^-)_4$	2.8813
	$^1(G^+/A^-)_5$	2.8819
	$^1(G^+/A^-)_6$	2.8959
	$^1G^*_1$	2.9111
	$^1G^*_2$	2.9764
	$^1G^*_3$	2.9800
	$^1(D^+/A^-)_1$	3.0178
	$^1(D^+/A^-)_2$	3.0205
^{1}(TCTA/Ir(ppy)$_3$/TPBI)	$^1(D^+/A^-)_1$	2.9082
	$^1G^*_1$	2.9132
	$^1(D^+/A^-)_2$	2.9190
	$^1G^*_2$	2.9780
	$^1G^*_3$	3.0049

[a] The optimized ω(CPCM) value in the presence of CPCM (ε_r = 3.0) is 0.023 bohr^{-1}.

Table S2. Triplet excited state energies obtained by TDA-TDDFT/CPCM and the ωB97X-D3 functional with the optimized ω(CPCM) value.[a]

		E (eV)
^{3}(Ir(ppy)$_3$/TCTA/TPBI)	$^3G^*_1$	2.7294
	$^3G^*_2$	2.7478
	$^3G^*_3$	2.7494
	$^3A^*_1$	2.8864
	$^3G^*_4$	2.9445
	$^3G^*_5$	2.9651
	$^3G^*_6$	2.9754
	$^3(D^+/A^-)_1$	2.9857
	$^3(D^+/A^-)_2$	2.9866
^{3}(TCTA/TPBI/Ir(ppy)$_3$)	$^3(G^+/A^-)_1$	2.6960
	$^3(G^+/A^-)_2$	2.6996
	$^3G^*_1$	2.7171
	$^3G^*_2$	2.7733
	$^3G^*_3$	2.7796
	$^3(G^+/A^-)_3$	2.8622
	$^3(G^+/A^-)_4$	2.8654
	$^3(G^+/A^-)_5$	2.8739
	$^3(G^+/A^-)_6$	2.8754
	$^3A^*_1$	2.8997
	$^3G^*_4$	2.9556
	$^3G^*_5$	2.9705
	$^3G^*_6$	2.9731
	$^3(D^+/A^-)_1$	3.0178
	$^3(D^+/A^-)_2$	3.0204

	$^3G^*_1$	2.7157
	$^3G^*_2$	2.7544
	$^3G^*_3$	2.7667
	$^3A^*_1$	2.8855
^{3}(TCTA/Ir(ppy)$_3$/TPBI)	$^3(D^+/A^-)_1$	2.9082
	$^3(D^+/A^-)_2$	2.9189
	$^3G^*_4$	2.9276
	$^3G^*_5$	2.9797
	$^3G^*_6$	2.9912

[a] The optimized ω(CPCM) value in the presence of CPCM (ε_r = 3.0) is 0.023 bohr^{-1}.

Table S3. Partial charges[a] on $Ir(ppy)_3$, TCTA, and TPBI (G, D, and A) for cohost CT states of three trimer model systems.

	$(Ir(ppy)_3/TCTA/TPBI)$			$(TCTA/TPBI/Ir(ppy)_3)$			$(TCTA/Ir(ppy)_3/TPBI)$		
	G	D	A	G	D	A	G	D	A
$^1(D^+/A^-)_1$	0.0004	0.9991	–0.9995	–0.0095	0.9997	–0.9902	–0.0002	0.9997	–0.9995
$^1(D^+/A^-)_2$	0.0004	0.9992	–0.9996	–0.0102	0.9995	–0.9893	0.0	0.9996	–0.9996
$^3(D^+/A^-)_1$	0.0004	0.9989	–0.9993	–0.0095	0.9992	–0.9897	–0.0002	0.9992	–0.9991
$^3(D^+/A^-)_2$	0.0004	0.9988	–0.9991	–0.0102	0.9989	–0.9887	0.0	0.9994	–0.9994

[a] In the unit of *e*.

Table S4. Electronic couplings[a] between singlet excited states for ^{1}(Ir(ppy)$_3$/TCTA/TPBI).

	$^1G^*_1$	$^1G^*_2$	$^1G^*_3$	$^1(D^+/A^-)_1$	$^1(D^+/A^-)_2$
$^1G^*_1$	-	–148	–152	12.4	0.13
$^1G^*_2$	–148	-	–133	–7.54	10.2
$^1G^*_3$	–152	–133	-	–3.96	–11.7
$^1(D^+/A^-)_1$	12.4	–7.54	–3.96	-	1.09
$^1(D^+/A^-)_2$	0.13	10.2	–11.7	1.09	-

[a] All electronic couplings are given in cm^{-1}.

Table S5. Electronic couplings[a] between singlet excited states for $^{1}(TCTA/TPBI/Ir(ppy)_3)$.

	$^{1}(G^+/A^-)_1$	$^{1}(G^+/A^-)_2$	$^{1}(G^+/A^-)_3$	$^{1}(G^+/A^-)_4$	$^{1}(G^+/A^-)_5$	$^{1}(G^+/A^-)_6$	$^{1}G^*_1$	$^{1}G^*_2$	$^{1}G^*_3$	$^{1}(D^+/A^-)_1$	$^{1}(D^+/A^-)_2$
$^{1}(G^+/A^-)_1$	-	–269	275	37.4	5.0	54.6	–27.4	28.4	51.9	–5.44	–1.02
$^{1}(G^+/A^-)_2$	–269	-	220	293	–96.3	–206	–162	401	–162	3.93	0.14
$^{1}(G^+/A^-)_3$	275	220	-	–126	–386	–183	209	180	349	–0.26	1.47
$^{1}(G^+/A^-)_4$	37.4	293	–126	-	44.8	341	–74.7	–5.93	78.1	4.18	–0.91
$^{1}(G^+/A^-)_5$	5.0	–96.3	–386	44.8	-	–282	–12.9	35.1	48.1	5.26	–2.02
$^{1}(G^+/A^-)_6$	54.6	–206	–183	341	–282	-	302	214	–209	2.77	0.99
$^{1}G^*_1$	–27.4	–162	209	–74.7	–12.9	302	-	148	144	–0.02	–0.46
$^{1}G^*_2$	28.4	401	180	–5.93	35.1	214	148	-	–137	–1.73	0.55
$^{1}G^*_3$	51.9	–162	349	78.1	48.1	–209	144	–137	-	–0.66	–0.76
$^{1}(D^+/A^-)_1$	–5.44	3.93	–0.26	4.18	5.26	2.77	–0.02	–1.73	–0.66	-	–0.85
$^{1}(D^+/A^-)_2$	–1.02	0.14	1.47	–0.91	–2.02	0.99	–0.46	0.55	–0.76	–0.85	-

[a] All electronic couplings are given in cm^{-1}.

Table S6. Electronic couplings[a] between singlet excited states for $^1(TCTA/Ir(ppy)_3/TPBI)$.

	$^1(D^+/A^-)_1$	$^1G^*_1$	$^1(D^+/A^-)_2$	$^1G^*_2$	$^1G^*_3$
$^1(D^+/A^-)_1$	-	–0.24	12	0.042	–0.061
$^1G^*_1$	–0.24	-	0.18	135	–144
$^1(D^+/A^-)_2$	12	0.18	-	0.18	–0.2
$^1G^*_2$	0.042	135	0.18	-	147
$^1G^*_3$	–0.061	–144	–0.2	147	-

[a] All electronic couplings are given in cm^{-1}.

Table S7. Electronic couplings[a] between triplet excited states for ^{3}(Ir(ppy)$_3$/TCTA/TPBI).

(*D*, *A*)	$^3G^*_1$	$^3G^*_2$	$^3G^*_3$	$^3A^*_1$	$^3G^*_4$	$^3G^*_5$	$^3G^*_6$	$^3(D^+/A^-)_1$	$^3(D^+/A^-)_2$
$^3G^*_1$	-	–29.2	–27.6	–0.0025	–612	–12.7	16.5	–0.02	0.065
$^3G^*_2$	–29.2	-	–30.1	–0.0011	11.3	–611	3.84	–0.015	0.0052
$^3G^*_3$	–27.6	–30.1	-	–0.00081	–17.8	19.9	–628	0.035	–0.036
$^3A^*_1$	–0.0025	–0.0011	–0.00081	-	–0.00075	0.0018	–0.00057	7.22	6.59
$^3G^*_4$	–612	11.3	–17.8	–0.00075	-	–56.9	–55.6	–0.019	0.052
$^3G^*_5$	–12.7	–611	19.9	0.0018	–56.9	-	–52.7	–0.0086	0.012
$^3G^*_6$	16.5	3.84	–628	–0.00057	–55.6	–52.7	-	0.0082	0.026
$^3(D^+/A^-)_1$	–0.02	–0.015	0.035	7.22	–0.019	–0.0086	0.0082	-	–0.069
$^3(D^+/A^-)_2$	0.065	0.0052	–0.036	6.59	0.052	0.012	0.026	–0.069	-

[a] All electronic couplings are given in cm^{-1}.

Table S8. Electronic couplings[a] between triplet excited states for ^{3}(TCTA/TPBI/Ir(ppy)$_3$).

(*D*, *A*)	$^3(G^+/A^-)_1$	$^3(G^+/A^-)_2$	$^3G^*_1$	$^3G^*_2$	$^3G^*_3$	$^3(G^+/A^-)_3$	$^3(G^+/A^-)_4$	$^3(G^+/A^-)_5$	$^3(G^+/A^-)_6$	$^3A^*_1$	$^3G^*_4$	$^3G^*_5$	$^3G^*_6$	$^3(D^+/A^-)_1$	$^3(D^+/A^-)_2$
$^3(G^+/A^-)_1$	-	–16	–177	7.8	–311	–15.4	366	158	312	–4.21	–24.2	–47.4	–52.7	–0.98	0.3
$^3(G^+/A^-)_2$	–16	-	129	300	8.28	130	–230	395	131	23.4	–9.5	51.8	–97.3	1.94	1.12
$^3G^*_1$	–177	129	-	65.9	75.6	–424	–183	–84.6	155	–2.02	–46	86.3	99.1	–0.81	0.14
$^3G^*_2$	7.8	300	65.9	-	32.8	16.2	66	28.2	103	0.17	298	24.7	33.4	–0.26	0.24
$^3G^*_3$	–311	8.28	75.6	32.8	-	159	–54.4	131	17.3	–11.1	7.92	–246	42.7	–0.075	–0.11
$^3(G^+/A^-)_3$	–15.4	130	–424	16.2	159	-	5.94	79.7	–76.2	–74.3	186	–248	–43.1	2.04	–0.9
$^3(G^+/A^-)_4$	366	–230	–183	66	–54.4	5.94	-	102	–196	–58	66	–84.8	–315	2.57	–1.35
$^3(G^+/A^-)_5$	158	395	–84.6	28.2	131	79.7	102	-	–72.4	2.71	248	20.9	–36.5	–0.25	–0.42
$^3(G^+/A^-)_6$	312	131	155	103	17.3	–76.2	–196	–72.4	-	43.2	–69.6	314	2.09	–0.016	–2.64
$^3A^*_1$	–4.21	23.4	–2.02	0.17	–11.1	–74.3	–58	2.71	43.2	-	5.04	–0.38	–6.77	–3.13	–5.14
$^3G^*_4$	–24.2	–9.5	–46	298	7.92	186	66	248	–69.6	5.04	-	47.3	60.5	–0.092	0.14
$^3G^*_5$	–47.4	51.8	86.3	24.7	–246	–248	–84.8	20.9	314	–0.38	47.3	-	–59.4	–0.12	0.049
$^3G^*_6$	–52.7	–97.3	99.1	33.4	42.7	–43.1	–315	–36.5	2.09	–6.77	60.5	–59.4	-	0.21	–0.046
$^3(D^+/A^-)_1$	–0.98	1.94	–0.81	–0.26	–0.075	2.04	2.57	–0.25	–0.016	–3.13	–0.092	–0.12	0.21	-	–5.11
$^3(D^+/A^-)_2$	0.3	1.12	0.14	0.24	–0.11	–0.9	–1.35	–0.42	–2.64	–5.14	0.14	0.049	–0.046	–5.11	-

[a] All electronic couplings are given in cm^{-1}.

Table S9. Electronic couplings[a] between triplet excited states for ^{3}(TCTA/Ir(ppy)$_3$/TPBI).

(*D*, *A*)	$^3G^*_1$	$^3G^*_2$	$^3G^*_3$	$^3A^*_1$	$^3(D^+/A^-)_1$	$^3(D^+/A^-)_2$	$^3G^*_4$	$^3G^*_5$	$^3G^*_6$
$^3G^*_1$	-	30.4	–27.8	0.063	–0.013	0.002	–594	19.2	–9.82
$^3G^*_2$	30.4	-	28.1	0.0025	–0.0033	–0.0028	–20	7.88	–630
$^3G^*_3$	–27.8	28.1	-	0.0037	–0.0014	0.00022	–8.05	–621	–15.9
$^3A^*_1$	0.063	0.0025	0.0037	-	–1.98	–0.3	–0.15	–0.00026	0.0031
$^3(D^+/A^-)_1$	–0.013	–0.0033	–0.0014	–1.98	-	11.6	0.0033	–0.0021	0.0022
$^3(D^+/A^-)_2$	0.002	–0.0028	0.00022	–0.3	11.6	-	0.0014	–0.00099	–0.00046
$^3G^*_4$	–594	–20	–8.05	–0.15	0.0033	0.0014	-	–58.8	54.1
$^3G^*_5$	19.2	7.88	–621	–0.00026	–0.0021	–0.00099	–58.8	-	55.4
$^3G^*_6$	–9.82	–630	–15.9	0.0031	0.0022	–0.00046	54.1	55.4	-

[a] All electronic couplings are given in cm^{-1}.

Table S10. Rate constants[a] for electronic transitions $i \rightarrow j$ by the Förster theory for ^{1}(Ir(ppy)$_3$/TCTA/TPBI).

$(i, j)^b$	$^1G^*_1$	$^1G^*_2$	$^1G^*_3$	$^1(D^+/A^-)_1$	$^1(D^+/A^-)_2$
$^1G^*_1$	-	1.96×10^{10}	1.80×10^{10}	1.18×10^{8}	1.28×10^{4}
$^1G^*_2$	1.94×10^{11}	-	4.74×10^{10}	1.49×10^{8}	2.70×10^{8}
$^1G^*_3$	2.28×10^{11}	6.07×10^{10}	-	4.65×10^{7}	3.97×10^{8}
$^1(D^+/A^-)_1$	1.55×10^{9}	1.98×10^{8}	4.82×10^{7}	-	3.52×10^{6}
$^1(D^+/A^-)_2$	1.74×10^{5}	3.71×10^{8}	4.25×10^{8}	3.64×10^{6}	-

[a] In s^{-1} units.
[b] *i* and *j* denote the donor (column) and acceptor (row) states for electronic transitions, respectively.

Table S11. Rate constants[a] for electronic transitions $i \rightarrow j$ by the Förster theory for ^{1}(TCTA/TPBI/Ir(ppy)$_3$).

(i, j)[b]	$^1(G^+/A^-)_1$	$^1(G^+/A^-)_2$	$^1(G^+/A^-)_3$	$^1(G^+/A^-)_4$	$^1(G^+/A^-)_5$	$^1(G^+/A^-)_6$	$^1G^*_1$	$^1G^*_2$	$^1G^*_3$	$^1(D^+/A^-)_1$	$^1(D^+/A^-)_2$
$^1(G^+/A^-)_1$	-	2.05×10^{11}	1.14×10^{10}	1.63×10^{8}	2.88×10^{6}	3.10×10^{8}	4.08×10^{7}	7.28×10^{6}	2.20×10^{7}	7.88×10^{4}	2.54×10^{3}
$^1(G^+/A^-)_2$	2.32×10^{11}	-	7.92×10^{9}	1.09×10^{10}	1.16×10^{9}	4.79×10^{9}	1.55×10^{9}	1.60×10^{9}	2.34×10^{8}	4.52×10^{4}	5.37×10^{1}
$^1(G^+/A^-)_3$	2.21×10^{12}	1.36×10^{12}	-	3.92×10^{10}	3.93×10^{11}	7.59×10^{10}	5.91×10^{10}	1.03×10^{10}	3.55×10^{10}	7.75×10^{3}	2.35×10^{5}
$^1(G^+/A^-)_4$	4.72×10^{10}	2.78×10^{12}	5.84×10^{10}	-	5.99×10^{9}	3.22×10^{11}	9.32×10^{9}	1.41×10^{7}	2.26×10^{9}	2.61×10^{6}	1.16×10^{5}
$^1(G^+/A^-)_5$	8.52×10^{8}	3.02×10^{11}	5.54×10^{11}	6.14×10^{9}	-	2.22×10^{11}	2.83×10^{8}	5.03×10^{8}	8.69×10^{8}	4.21×10^{6}	5.81×10^{5}
$^1(G^+/A^-)_6$	1.07×10^{11}	1.46×10^{12}	1.35×10^{11}	3.85×10^{11}	2.60×10^{11}	-	1.67×10^{11}	2.05×10^{10}	1.80×10^{10}	1.28×10^{6}	1.53×10^{5}
$^1G^*_1$	3.73×10^{10}	1.26×10^{12}	2.79×10^{11}	2.95×10^{10}	8.76×10^{8}	4.43×10^{11}	-	1.73×10^{10}	1.51×10^{10}	1.21×10^{2}	6.09×10^{4}
$^1G^*_2$	8.33×10^{10}	1.61×10^{13}	6.07×10^{11}	5.60×10^{8}	1.95×10^{10}	6.79×10^{11}	2.16×10^{11}	-	5.27×10^{10}	3.91×10^{6}	3.79×10^{5}
$^1G^*_3$	2.89×10^{11}	2.73×10^{12}	2.41×10^{12}	1.03×10^{11}	3.86×10^{10}	6.87×10^{11}	2.17×10^{11}	6.05×10^{10}	-	6.17×10^{5}	7.71×10^{5}
$^1(D^+/A^-)_1$	4.48×10^{9}	2.27×10^{9}	2.27×10^{6}	5.13×10^{8}	8.08×10^{8}	2.11×10^{8}	7.52×10^{3}	1.94×10^{7}	2.66×10^{6}	-	2.07×10^{6}
$^1(D^+/A^-)_2$	1.60×10^{8}	2.99×10^{6}	7.62×10^{7}	2.54×10^{7}	1.24×10^{8}	2.80×10^{7}	4.19×10^{6}	2.09×10^{6}	3.70×10^{6}	2.30×10^{6}	-

[a] In s^{-1} units.

[b] i and j denote the donor (column) and acceptor (row) states for electronic transitions, respectively.

Table S12. Rate constants[a] for electronic transitions $i \rightarrow j$ by the Förster theory for ^{1}(TCTA/Ir(ppy)$_3$/TPBI).

(i, j)[b]	$^1(D^+/A^-)_1$	$^1G^*_1$	$^1(D^+/A^-)_2$	$^1G^*_2$	$^1G^*_3$
$^1(D^+/A^-)_1$	-	1.56×10^5	3.50×10^8	1.24×10^3	1.45×10^3
$^1G^*_1$	1.89×10^5	-	8.59×10^4	1.44×10^{10}	8.97×10^9
$^1(D^+/A^-)_2$	5.31×10^8	1.07×10^5	-	2.78×10^4	1.99×10^4
$^1G^*_2$	1.85×10^4	1.76×10^{11}	2.73×10^5	-	3.83×10^{10}
$^1G^*_3$	6.13×10^4	3.11×10^{11}	5.52×10^5	1.08×10^{11}	-

[a] In s^{-1} units.
[b] *i* and *j* denote the donor (column) and acceptor (row) states for electronic transitions, respectively.

Table S13. Rate constants[a] for electronic transitions $i \rightarrow j$ by the Förster theory for ^{3}(Ir(ppy)$_3$/TCTA/TPBI).

(i, j)[b]	$^3G^*_1$	$^3G^*_2$	$^3G^*_3$	$^3A^*_1$	$^3G^*_4$	$^3G^*_5$	$^3G^*_6$	$^3(D^+/A^-)_1$	$^3(D^+/A^-)_2$
$^3G^*_1$	-	1.80×10^{9}	1.56×10^{9}	5.61×10^{-1}	7.16×10^{9}	1.72×10^{6}	2.18×10^{6}	2.46×10^{0}	2.42×10^{1}
$^3G^*_2$	3.66×10^{9}	-	2.65×10^{9}	1.58×10^{-1}	4.05×10^{6}	6.71×10^{9}	1.99×10^{5}	2.39×10^{0}	2.63×10^{-1}
$^3G^*_3$	3.37×10^{9}	2.82×10^{9}	-	9.76×10^{-2}	1.04×10^{7}	7.46×10^{6}	5.56×10^{9}	1.28×10^{1}	1.31×10^{1}
$^3A^*_1$	2.44×10^{2}	3.36×10^{1}	1.95×10^{1}	-	5.12×10^{-1}	1.85×10^{0}	1.51×10^{-1}	1.90×10^{7}	1.55×10^{7}
$^3G^*_4$	2.94×10^{13}	8.16×10^{9}	1.98×10^{10}	4.84×10^{0}	-	6.51×10^{9}	5.04×10^{9}	4.91×10^{2}	3.50×10^{3}
$^3G^*_5$	1.57×10^{10}	3.00×10^{13}	3.14×10^{10}	3.88×10^{1}	1.44×10^{10}	-	6.85×10^{9}	1.49×10^{2}	3.02×10^{2}
$^3G^*_6$	2.95×10^{10}	1.32×10^{9}	3.48×10^{13}	4.71×10^{0}	1.66×10^{10}	1.02×10^{10}	-	1.65×10^{2}	1.67×10^{3}
$^3(D^+/A^-)_1$	4.97×10^{4}	2.38×10^{4}	1.19×10^{5}	8.86×10^{8}	2.42×10^{3}	3.30×10^{2}	2.45×10^{2}	-	1.41×10^{4}
$^3(D^+/A^-)_2$	5.07×10^{5}	2.70×10^{3}	1.26×10^{5}	7.49×10^{8}	1.79×10^{4}	6.93×10^{2}	2.58×10^{3}	1.46×10^{4}	-

[a] In s^{-1} units.

[b] i and j denote the donor (column) and acceptor (row) states for electronic transitions, respectively.

Table S14. Rate constants[a] for electronic transitions $i \rightarrow j$ by the Förster theory for ^{3}(TCTA/TPBI/Ir(ppy)$_3$).

(i, j)[b]	$^{3}(G^{+}/A^{-})_{1}$	$^{3}(G^{+}/A^{-})_{2}$	$^{3}G^{*}_{1}$	$^{3}G^{*}_{2}$	$^{3}G^{*}_{3}$	$^{3}(G^{+}/A^{-})_{3}$	$^{3}(G^{+}/A^{-})_{4}$	$^{3}(G^{+}/A^{-})_{5}$	$^{3}(G^{+}/A^{-})_{6}$	$^{3}A^{*}_{1}$	$^{3}G^{*}_{4}$	$^{3}G^{*}_{5}$	$^{3}G^{*}_{6}$	$^{3}(D^{+}/A^{-})_{1}$	$^{3}(D^{+}/A^{-})_{2}$
$^{3}(G^{+}/A^{-})_{1}$	-	7.18×10^{8}	6.25×10^{10}	3.67×10^{7}	5.08×10^{10}	1.67×10^{7}	8.75×10^{9}	1.30×10^{9}	4.90×10^{9}	4.64×10^{5}	3.12×10^{6}	7.71×10^{6}	8.82×10^{6}	7.57×10^{2}	6.57×10^{1}
$^{3}(G^{+}/A^{-})_{2}$	8.25×10^{8}	-	3.54×10^{10}	5.89×10^{10}	3.89×10^{7}	1.31×10^{9}	3.77×10^{9}	8.95×10^{9}	9.45×10^{8}	1.59×10^{7}	5.38×10^{5}	1.03×10^{7}	3.36×10^{7}	3.35×10^{3}	1.03×10^{3}
$^{3}G^{*}_{1}$	1.41×10^{11}	6.96×10^{10}	-	4.16×10^{9}	4.78×10^{9}	2.17×10^{10}	3.73×10^{9}	6.46×10^{8}	2.08×10^{9}	1.88×10^{5}	2.09×10^{7}	4.79×10^{7}	5.85×10^{7}	1.01×10^{3}	2.69×10^{1}
$^{3}G^{*}_{2}$	7.30×10^{8}	1.02×10^{12}	3.66×10^{10}	-	2.87×10^{9}	1.22×10^{8}	1.88×10^{9}	2.81×10^{8}	3.66×10^{9}	5.72×10^{3}	4.14×10^{9}	1.90×10^{7}	3.24×10^{7}	5.70×10^{2}	4.55×10^{2}
$^{3}G^{*}_{3}$	1.29×10^{12}	8.58×10^{8}	5.36×10^{10}	3.66×10^{9}	-	1.35×10^{10}	1.47×10^{9}	7.06×10^{9}	1.18×10^{8}	2.74×10^{7}	3.44×10^{6}	2.24×10^{9}	6.29×10^{7}	5.59×10^{1}	1.08×10^{2}
$^{3}(G^{+}/A^{-})_{3}$	1.04×10^{10}	7.05×10^{11}	5.95×10^{12}	3.80×10^{9}	3.29×10^{11}	-	1.00×10^{8}	1.53×10^{10}	1.35×10^{10}	7.86×10^{9}	1.45×10^{10}	1.82×10^{10}	5.18×10^{8}	3.88×10^{5}	7.10×10^{4}
$^{3}(G^{+}/A^{-})_{4}$	6.13×10^{12}	2.30×10^{12}	1.15×10^{12}	6.63×10^{10}	4.07×10^{10}	1.13×10^{8}	-	2.66×10^{10}	9.57×10^{10}	5.11×10^{9}	1.96×10^{9}	2.29×10^{9}	2.98×10^{10}	6.64×10^{5}	1.72×10^{5}
$^{3}(G^{+}/A^{-})_{5}$	1.27×10^{12}	7.59×10^{12}	2.78×10^{11}	1.38×10^{10}	2.71×10^{11}	2.40×10^{10}	3.70×10^{10}	-	1.54×10^{10}	1.33×10^{7}	3.35×10^{10}	1.71×10^{8}	4.88×10^{8}	8.00×10^{3}	2.07×10^{4}
$^{3}(G^{+}/A^{-})_{6}$	5.06×10^{12}	8.49×10^{11}	9.50×10^{11}	1.90×10^{11}	4.82×10^{9}	2.26×10^{10}	1.41×10^{11}	1.63×10^{10}	-	3.49×10^{9}	2.73×10^{9}	3.97×10^{10}	1.66×10^{6}	3.28×10^{1}	8.45×10^{5}
$^{3}A^{*}_{1}$	1.23×10^{9}	3.65×10^{10}	2.19×10^{8}	7.60×10^{5}	2.85×10^{9}	3.35×10^{10}	1.93×10^{10}	3.60×10^{7}	8.93×10^{9}	-	2.45×10^{7}	1.01×10^{5}	3.02×10^{7}	2.30×10^{6}	5.83×10^{6}
$^{3}G^{*}_{4}$	7.18×10^{10}	1.07×10^{10}	2.12×10^{11}	4.78×10^{12}	3.12×10^{9}	5.37×10^{11}	6.42×10^{10}	7.90×10^{11}	6.09×10^{10}	2.13×10^{8}	-	5.04×10^{9}	7.84×10^{9}	7.17×10^{3}	1.53×10^{4}
$^{3}G^{*}_{5}$	3.15×10^{11}	3.66×10^{11}	8.65×10^{11}	3.91×10^{10}	3.60×10^{12}	1.20×10^{12}	1.34×10^{11}	7.16×10^{9}	1.57×10^{12}	1.57×10^{6}	8.96×10^{9}	-	1.01×10^{10}	1.78×10^{4}	2.62×10^{3}
$^{3}G^{*}_{6}$	3.99×10^{11}	1.32×10^{12}	1.17×10^{12}	7.36×10^{10}	1.12×10^{11}	3.78×10^{10}	1.92×10^{12}	2.26×10^{10}	7.27×10^{7}	5.16×10^{8}	1.54×10^{10}	1.12×10^{10}	-	5.35×10^{4}	2.48×10^{3}
$^{3}(D^{+}/A^{-})_{1}$	1.93×10^{8}	7.43×10^{8}	1.14×10^{8}	7.30×10^{6}	5.61×10^{5}	1.59×10^{8}	2.41×10^{8}	2.09×10^{6}	8.09×10^{3}	2.22×10^{8}	7.95×10^{4}	1.11×10^{5}	3.01×10^{5}	-	7.49×10^{7}
$^{3}(D^{+}/A^{-})_{2}$	1.85×10^{7}	2.53×10^{8}	3.35×10^{6}	6.44×10^{6}	1.20×10^{6}	3.23×10^{7}	6.90×10^{7}	5.97×10^{6}	2.30×10^{8}	6.21×10^{8}	1.88×10^{5}	1.80×10^{4}	1.55×10^{4}	8.28×10^{7}	-

[a] In s^{-1} units.

[b] *i* and *j* denote the donor (column) and acceptor (row) states for electronic transitions, respectively.

Table S15. Rate constants[a] for electronic transitions $i \rightarrow j$ by the Förster theory for ^{3}(TCTA/Ir(ppy)$_3$/TPBI).

(i, j)[b]	$^3G^*_1$	$^3G^*_2$	$^3G^*_3$	$^3A^*_1$	$^3(D^+/A^-)_1$	$^3(D^+/A^-)_2$	$^3G^*_4$	$^3G^*_5$	$^3G^*_6$
$^3G^*_1$	-	1.29×10^{9}	8.29×10^{8}	2.53×10^{2}	6.45×10^{0}	1.06×10^{-1}	7.38×10^{9}	1.74×10^{6}	3.22×10^{5}
$^3G^*_2$	5.74×10^{9}	-	1.88×10^{9}	1.07×10^{0}	1.04×10^{0}	5.75×10^{-1}	2.37×10^{7}	8.92×10^{5}	4.12×10^{9}
$^3G^*_3$	5.96×10^{9}	3.03×10^{9}	-	3.17×10^{0}	2.78×10^{-1}	4.86×10^{-3}	5.27×10^{6}	7.82×10^{9}	3.73×10^{6}
$^3A^*_1$	1.81×10^{5}	1.71×10^{2}	3.14×10^{2}	-	7.56×10^{6}	1.36×10^{5}	3.05×10^{4}	2.84×10^{-2}	2.97×10^{0}
$^3(D^+/A^-)_1$	1.11×10^{4}	3.99×10^{2}	6.63×10^{1}	1.82×10^{7}	-	3.28×10^{8}	2.29×10^{1}	2.98×10^{0}	2.50×10^{0}
$^3(D^+/A^-)_2$	2.74×10^{2}	3.33×10^{2}	1.75×10^{0}	4.96×10^{5}	4.96×10^{8}	-	4.88×10^{0}	8.46×10^{-1}	1.43×10^{-1}
$^3G^*_4$	2.68×10^{13}	1.92×10^{10}	2.66×10^{9}	1.55×10^{5}	4.86×10^{1}	6.83×10^{0}	-	3.62×10^{9}	2.39×10^{9}
$^3G^*_5$	4.74×10^{10}	5.44×10^{9}	2.96×10^{13}	1.09×10^{0}	4.74×10^{1}	8.89×10^{0}	2.72×10^{10}	-	7.39×10^{9}
$^3G^*_6$	1.37×10^{10}	3.92×10^{13}	2.20×10^{10}	1.77×10^{2}	6.21×10^{1}	2.35×10^{0}	2.79×10^{10}	1.15×10^{10}	-

[a] In s^{-1} units.
[b] i and j denote the donor (column) and acceptor (row) states for electronic transitions, respectively.

Table S16. Electronic Hamiltonian[a,b] adopted as the simplified 4-state system for the mixed quantum-classical simulations, extracted from ^{3}(TCTA/TPBI/Ir(ppy)$_3$).

	$^3(G^+/A^-)$	$^3G^*$	$^3A^*$	$^3(D^+/A^-)$
$^3(G^+/A^-)$	0.0	–311	–74	–2.64
$^3G^*$	–311	170	–11	–0.81
$^3A^*$	–74	–11	1643	–5.14
$^3(D^+/A^-)$	–2.64	–0.81	–5.14	2595

[a] In cm^{-1} units.
[b] The diagonal and the off-diagonal elements of the Hamiltonian are excitation energies and electronic couplings, respectively.

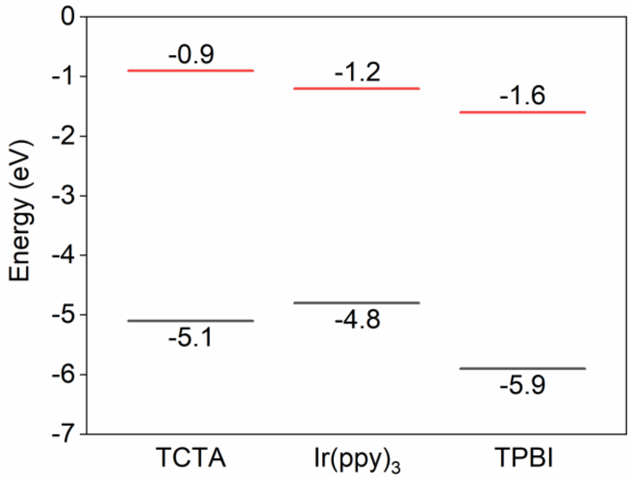

Figure S1. Energy level diagram showing the HOMO (black lines) and the LUMO (red lines) levels for TCTA, $Ir(ppy)_3$, and TPBI materials. The HOMO-LUMO gap of the (TCTA, TPBI) pair is larger than that of ($Ir(ppy)_3$, TPBI) pair, but smaller than that of (TCTA, $Ir(ppy)_3$) pair. The HOMO and LUMO energies were obtained from DFT calculations with the B3LYP functional.

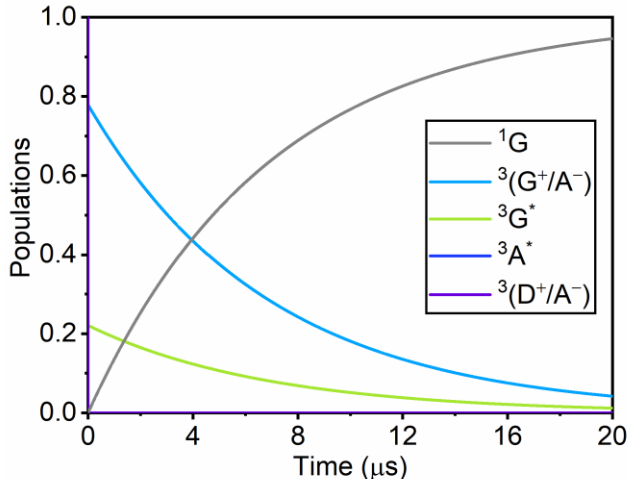

Figure S2. Population changes in $^3(TCTA/TPBI/Ir(ppy)_3)$ with the assumption that the initial state is the lowest $^3(D^+/A^-)$ state. The figure is an extension of Figure 9b into the long-time limit. One can see that the population ratio between $^3(G^+/A^-)$ and $^3G^*$ is the same all the time due to their fast equilibration, and the two populations eventually decay to zero through the emissive relaxation of $^3G^* \rightarrow {}^1G$.

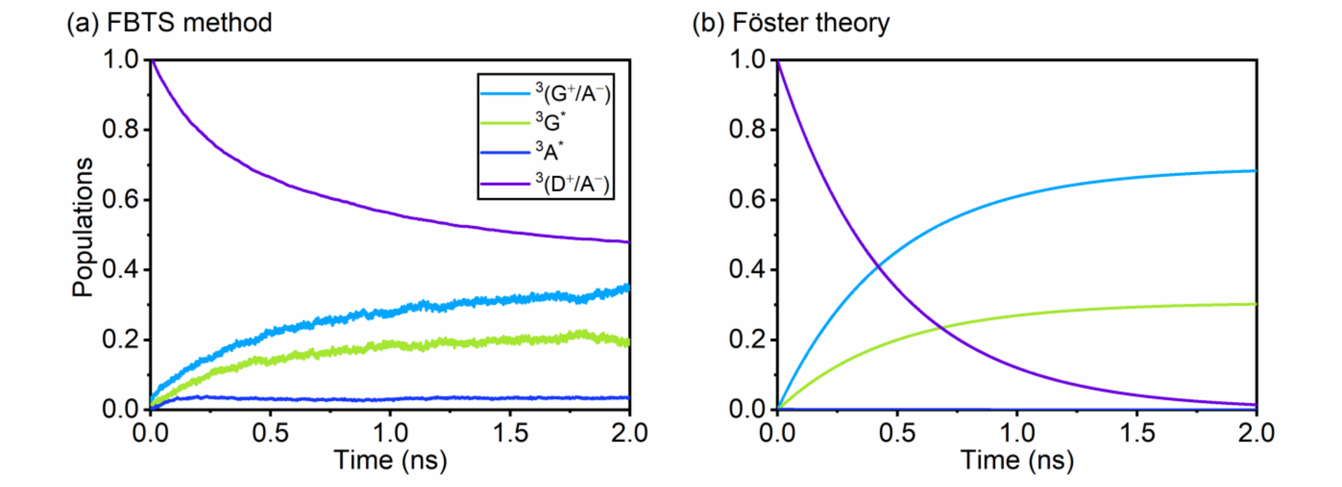

Figure S3. (a) Population dynamics is from the mixed quantum-classical (MQC) simulations with the forward-backward trajectory solution (FBTS) method [1,2]. We employed a simplified 4-state system, based on $^3(G^+/A^-)$, $^3G^*$, $^3A^*$, and $^3(D^+/A^-)$, as represented by the Hamiltonian given in Table S16. A total of 10,000 trajectories were employed with a time step of 0.5 fs. Influence of the environment was modeled by a Debye spectral density, $J(\omega) = 2\lambda_{\mathrm{ph}}\omega\omega_c / (\omega^2 + \omega_c^2)$ with $\lambda_{\mathrm{ph}} = 2000\,\mathrm{cm}^{-1}$ and $\omega_c = 300\,\mathrm{fs}$. This spectral density was implemented with 1000 discrete bath modes with a maximum frequency at $\omega_{\mathrm{max}} = 3000\,\mathrm{cm}^{-1}$. The MQC simulation result corresponds quite well with the results from the master equation formalism using the Förster theory, shown in (b).

Supporting References

1. Hsieh, C.-Y.; Kapral, R., Analysis of the forward-backward trajectory solution for the mixed quantum-classical Liouville equation. *J. Chem. Phys.* **2013**, *138*, 134110.

2. Hsieh, C.-Y.; Kapral, R., Nonadiabatic dynamics in open quantum-classical systems: Forward-backward trajectory solution. *J. Chem. Phys.* **2012**, *137*, 22A507.
